# Changes in plasma lipidome following initiation of antiretroviral therapy

**DOI:** 10.1371/journal.pone.0202944

**Published:** 2018-08-29

**Authors:** Janine M. Trevillyan, Gerard Wong, Rebekah Puls, Kathy Petoumenos, Sean Emery, Natalie A. Mellett, Piyushkumar A. Mundra, Peter J. Meikle, Jennifer F. Hoy, for the ALTAIR Study Group

**Affiliations:** 1 Department of Infectious Diseases, Faculty of Medicine, Nursing and Health Science, Monash University, Melbourne, Australia; 2 Baker Heart and Diabetes Institute, Melbourne, Australia; 3 Faculty of Medicine, UNSW Sydney, Sydney, Australia; 4 The Kirby Institute, UNSW Sydney, Sydney, Australia; 5 Faculty of Medicine, University of Queensland, Brisbane, Australia; 6 Department of Infectious Diseases, Alfred Hospital, Melbourne, Australia; Harvard Medical School, UNITED STATES

## Abstract

**Introduction:**

HIV and antiretroviral therapy (ART) have been associated with increased cardiovascular disease and important changes in lipid metabolism. Advances in mass-spectrometry technology allow for the detailed assessment of individual lipid species which may illuminate the mechanisms underlying increased cardiovascular risk. We describe the change in plasma lipidome with initiation of antiretroviral therapy and compare these by regimen.

**Methods:**

Plasma lipid profiling (by electrospray isonisation-tandem mass spectrometry) was performed on ARV-naive HIV positive participants randomised to one of three regimens; tenofovir/emtricitabine with efavirenz, ritonavir-boosted atazanavir (atazanavir/r) or zidovudine/abacavir. Participants (n = 115) who remained on their randomised regimen with complete samples available at baseline, week 12 and 48 were included. 306 lipid species from 22 lipid classes were analysed.

**Results:**

Initiation of ART led to significant changes in lipidome which were partly dependent on the randomised regimen received. This led to significant differences in 72 lipid species and 7 classes (cholesterol ester, free cholesterol, phosphatidylcholine, GM3 ganglioside, trihexosylceramide, monohexosylceramide, and ceramides) by arm at week 48. Consistently higher lipid concentrations were seen with efavirenz compared with atazanavir/r or zidovudine/abacavir. Twelve of the lipid species and two lipid classes (cholesterol esters and ceramides) that were significantly increased in the efavirenz arm compared with the atazanavir/r or zidovudine/abacavir arms have previously been associated with future cardiovascular events in HIV positive patients. Change in HIV viral load was predictive of change in 3 lipid species.

**Conclusions:**

Initiation of ART lead to significant changes in the plasma lipidome that were greatest in those receiving efavirenz.

## Introduction

HIV infection and the use of antiretroviral (ARV) therapy (ART) is associated with an increased risk for cardiovascular disease (CVD) [1]. HIV and ARV-associated dyslipidaemia is responsible for a component of this elevated risk. Following HIV infection, prior to initiation of ART, total, low-density lipoprotein (LDL) and high- density lipoprotein (HDL) cholesterol frequently decline [2], while initiation of ART commonly leads to increases in triglycerides, total and LDL cholesterol but only modest, if any, correction in HDL [3]. This pattern of lipid abnormalities (elevated total- and LDL- cholesterol with depressed HDL) often referred to as “mixed-dyslipidaemia” has been shown in the general population to be associated with the development and progression of atherosclerosis [4]. Yet predictions of cardiovascular risk based on these measures which are useful in the general population often underestimate the degree of risk in HIV positive individuals and thus novel methods to predict CVD risk in the setting of HIV are critical for optimal assessment and management [5].

Advances in soft-ionization mass spectrometry allow for the rapid and sensitive detection of a large variety of lipid species from small volume samples based on their molecular mass after fragmentation [6]. Thus a detailed ‘lipid profile’ can be created from an individual sample. Much like the field of genomics which has provided a deeper understanding of the impact genetic differences have on human diseases and drug effectiveness, lipidomics (the analysis and characterisation of lipid species and their interactions) uses novel technology to shed new light on a longstanding medical conundrum. More than simple structural building blocks, lipids, both cell bound and in plasma, play a crucial role in multiple organ functions. Lipids constitute a key component of atherosclerotic plaque, both as structural elements within fatty streaks and foam cells and also as triggers which potentiate ongoing inflammatory responses [7]. Individual lipid species are functionally distinct and differ substantially in their capacity and propensity to play an atheroprotective or atherogenic role. Thus any physiological or pharmacological factors which disturb lipid homeostasis at an individual lipid level could greatly alter the progression of CVD.

Analysis of plasma lipidomes have identified previously undetectable, highly significant differences in lipid species and classes between HIV positive and negative participants as well as between individuals with and without coronary artery disease (CAD) [8]. This technology may allow for a more precise understanding of the impact HIV and ART have on lipid homeostasis and cardiovascular risk. Previously it has been hypothesised that the elevated cardiovascular risk associated with ART which persists despite correction for fasting lipid levels is the consequence of inflammation and immune activation associated with chronic HIV, but it is plausible that some of this effect may be the result of changes in plasma lipidome. In this study we aimed to comprehensively identify and contrast the effects initiation of different ARV regimens have on the plasma lipidome.

## Materials and methods

Lipidomic analysis was performed on a subset of ARV-naïve HIV positive individuals who were randomised to their initial ARV regimen as part of the ALTernative Antiretroviral strategies: a comparison of three Initial Regimens (ALTAIR) trial (ClinicalTrials.gov number, NCT00335322). A detailed description of the methods and results of the primary study have been described elsewhere [9]. In brief, ALTAIR was an international randomised, open label trial comparing three initial ARV regimens. The three arms consisted of co-formulated tenofovir disoproxil fumurate (DF)-emtricitabine with efavirenz (Arm I), ritonavir-boosted atazanavir (atazanavir/r) (Arm II) or zidovudine/abacavir (Arm III), with the primary endpoint of non-inferior antiretroviral efficacy at 48 weeks. The novel quadruple nucleo(t)side combination (Arm III) of the ALTAIR study demonstrated significantly less suppression of HIV replication compared with that of the standard ARV regimens. This post hoc study utilised stored samples collected at baseline (ARV naïve) and at 12 and 48 weeks after initiation of ART. Participants who remained on the randomised treatment allocation and had stored samples available at all three time points were included.

### Lipidomic analysis

Lipid profiling was performed as previously described [10]. In brief, samples were randomised and processed (including laboratory controls) in a single day to minimise variability. Lipid classes were calculated as the sum of the individual lipid species within each class. A total of 331 lipid species distributed across 24 lipid classes were initially analysed. 25 species and 2 classes were found to be in very low abundance and were thus excluded as their reported measurements were deemed to be unreliable. Hence 306 lipid species from 22 classes were included in the final analysis. A full description of the experimental protocol and list of the lipid species and classes analysed are available in S1 File.

### Statistical analyses

Baseline participant characteristics were described using medians and interquartile ranges (IQR) for continuous variables and frequencies and percentages for categorical variables. Results were summarised by treatment allocation and the chi-square test was used to evaluate the difference in proportions of the arms for categorical variables and the Mann-Whitney U-test used to evaluate the difference in the medians of the arms for continuous variables.

Multiple linear regression was applied to examine the association of plasma lipid species with HIV specific factors (CD4+ T cell count, CD4+ T cell nadir count and HIV viral load) prior to commencing ART following adjustment for age, gender, body mass index (BMI) and systolic blood pressure (SBP). Multiple linear regression was also applied to examine the association of conventional lipids (total cholesterol, HDL, LDL, triglycerides) with lipid species and classes (adjusted as above).

The difference in lipid species concentration between treatment arms was assessed using the Kruskal-Wallis test with post-hoc comparisons. Results were considered statistically significant when p <0.05 following adjustment for multiple comparisons (using the Benjamini-Hochberg method for Kruskal-Wallis tests and the Dunn-Sidak correction for post-hoc tests) [11].

The ALTAIR trial was approved by all local ethics committees and participants provided informed consent for blood samples to be stored for use in future studies. This substudy received approval from the Alfred Hospital Ethics Committee (Project Number: 423/12).

## Results

Baseline participant characteristics (n = 115, 32 efavirenz arm; 44 atazanavir/r arm; 39 zidovudine/abacavir arm) are described in Table 1. Overall 95 (82.6%) were male with a median age of 36.9 years (inter-quartile range [IQR] 29.78, 41.96). Participants included in this substudy were representative of the original clinical trial population as a whole with no statistically significant differences in characteristics (data not shown). There were no differences in baseline characteristics (including HIV specific and CVD risk factors) between the three arms, and specifically no differences between baseline lipid measurements (including all lipid classes and species) across the three treatment allocations.

**Table 1:** Baseline participant characteristics. Median and interquartile range unless otherwise stated, all arms received tenofovir disoproxil fumurate and emtricitabine in combination with the third agent.

	Arm I (Efavirenz)	Arm II (Atazanavir/r)	Arm III (Zidovudine/abacavir)
**n**	32	44	39
**Male,** n (%)	26 (81.2)	36 (81.8)	33 (84.6)
**Age**, years	38.4 (32.0–42.6)	37.1 (28.4–41.3)	34.9 (29.1–43.3)
**Current Smoker,** n (%)	17 (53.1)	16 (36.3)	20 (51.2)
**Systolic BP,** mmHg	120 (111–126)	120 (111–125)	120 (110–120)
**BMI,** kg/m^2^	23.3 (21.1–25.4)	23.4 (21.1–25.6)	23.2 (21.1–25.0)
**Glucose,** mmol/l	4.84 (4.68–5.11)	4.76 (4.57–5.21)	4.77 (4.40–5.40)
**Cholesterol,** mmol/l	4.43 (3.69–4.90)	3.77 (3.15–4.45)	3.76 (3.30–4.32)
**HDL,** mmol/l	0.89 (0.80–1.04)	0.85 (0.70–1.00)	0.90 (0.73–1.04)
**LDL,** mmol/l	2.54 (2.21–3.28)	2.15 (1.71–2.71)	2.33 (1.90–2.87)
**Triglycerides,** mmol/l	1.31 (1.00–2.04)	1.18 (0.92–1.95)	1.02 (0.90–1.40)
**On statin therapy**	0 (0.0)	2 (4.5)	1 (2.5)
**HIV Duration,** years	1.3 (0.9–3.0)	1.7 (0.8–4.1)	2.1 (0.6–3.7)
**HIV Viral load,** copies/ml	36,800 (12,111->100,000)	92,637 (30,100->100,000)	56,100 (11,707->100,000)
**CD4+ T cell count,** cells/μL	217 (173–265)	210 (126–318)	206 (132–268)
**CD4+ T cell Nadir,** cells/μL	206 (149–243)	174 (119–260)	179 (116–220)

Abbreviations: Blood Pressure (**BP**); Body Mass Index (**BMI**); High-density lipoprotein (**HDL**); Low-density lipoprotein (**LDL**)

### Association of baseline characteristics with the plasma lipidome

Following adjustment for age, gender, BMI and SBP there was a strong association between baseline total cholesterol and LDL level with the baseline plasma lipidome (total cholesterol: 19 lipid classes and 213 individual lipid species significantly associated; LDL: 13 lipid classes and 167 lipid species significant associated) (see Table 2). Fewer lipid classes were associated with baseline HDL (9 classes; 70 species) or triglycerides (7 classes; 128 species).

**Table 2:** Association between baseline cholesterol measurements and lipid classes. Linear regression analysis, corrected by Benjamini-Hochberg method and adjusted for age, sex, body mass index, and systolic blood pressure.

Lipid Class	Total Cholesterol		LDL		HDL		Triglycerides	
	β co-efficient (95% CI)	*p-value*	β co-efficient (95% CI)	*p-value*	β co-efficient (95% CI)	*p-value*	β co-efficient (95% CI)	*p-value*
**Ceramide (Cer)**	0.63 (0.46, 0.80)	**<0.001**	0.45 (0.29, 0.61)	**<0.001**	0.05 (0.00, 0.11)	0.112	0.23 (0.06, 0.41)	**0.029**
**Monohexosylceramide (MHC)**	0.40 (0.21, 0.59)	**<0.001**	0.34 (0.17, 0.51)	**<0.001**	0.04 (-0.02, 0.10)	0.216	0.03 (-0.15, 0.20)	0.778
**Dihexosylceramide (DHC)**	0.32 (0.11, 0.53)	**0.005**	0.35 (0.17, 0.53)	**<0.001**	0.04 (-0.02, 0.10)	0.216	-0.17 (-0.36, 0.01)	0.171
**Trihexosylceramide (THC)**	0.25 (0.09, 0.41)	**0.005**	0.22 (0.08, 0.36)	**0.004**	0.07 (0.03, 0.12)	**0.018**	-0.11 (-0.25, 0.03)	0.217
**GM3 ganglioside (GM3)**	0.59 (0.40, 0.79)	**<0.001**	0.50 (0.33, 0.67)	**<0.001**	0.03 (-0.03, 0.09)	0.433	0.11 (-0.08, 0.30)	0.321
**Sphingomyelin (SM)**	0.62 (0.43, 0.82)	**<0.001**	0.48 (0.30, 0.65)	**<0.001**	0.09 (0.03, 0.15)	**0.018**	0.09 (-0.11, 0.28)	0.445
**Phosphatidylcholine (PC)**	0.55 (0.38, 0.72)	**<0.001**	0.34 (0.18, 0.50)	**<0.001**	0.08 (0.02, 0.13)	**0.022**	0.26 (0.10, 0.42)	**0.007**
**Alkylphosphatidylcholine (PC-O)**	0.39 (0.20, 0.57)	**<0.001**	0.37 (0.21, 0.53)	**<0.001**	0.05 (0.00, 0.10)	0.122	-0.10 (-0.27, 0.07)	0.321
**Phosphatidylcholine plasmalogen (PC-P)**	0.49 (0.30, 0.69)	**<0.001**	0.43 (0.26, 0.59)	**<0.001**	0.08 (0.03, 0.14)	**0.022**	-0.08 (-0.26, 0.10)	0.445
**Lysophosphatidylcholine (LPC)**	0.23 (0.04, 0.42)	**0.022**	0.09 (-0.08, 0.25)	0.384	0.07 (0.02, 0.12)	**0.032**	0.14 (-0.02, 0.30)	0.189
**Lysoalkylphosphatidylcholine (LPC(O))**	0.19 (-0.01, 0.39)	0.071	0.15 (-0.03, 0.32)	0.152	0.05 (0.00, 0.11)	0.096	-0.05 (-0.23, 0.12)	0.591
**Phosphatidylethanolamine (PE)**	0.19 (0.00, 0.37)	0.053	0.03 (-0.13, 0.19)	0.820	0.03 (-0.02, 0.08)	0.283	0.26 (0.11, 0.42)	**0.004**
**Akylphosphatidylethanolamine (PE-O)**	0.31 (0.15, 0.47)	**<0.001**	0.23 (0.09, 0.37)	**0.003**	0.08 (0.03, 0.12)	**0.018**	-0.04 (-0.18, 0.11)	0.639
**Phosphatidylethanolamine plasmalogen (PE-P)**	0.19 (0.04, 0.34)	**0.022**	0.18 (0.04, 0.31)	**0.016**	0.05 (0.00, 0.09)	0.072	-0.09 (-0.22, 0.05)	0.315
**Lysophosphatidylethanolamine (LPE)**	0.22 (0.02, 0.41)	**0.035**	0.09 (-0.08, 0.26)	0.384	0.07 (0.01, 0.12)	**0.035**	0.12 (-0.05, 0.29)	0.255
**Phosphatidylinositol (PI)**	0.52 (0.35, 0.69)	**<0.001**	0.32 (0.16, 0.48)	0.000	0.06 (0.00, 0.11)	**0.072**	0.28 (0.12, 0.44)	**0.004**
**Lysophosphatidylinositol (LPI)**	0.23 (0.06, 0.39)	**0.013**	0.08 (-0.06, 0.23)	0.367	0.06 (0.02, 0.11)	**0.023**	0.13 (-0.02, 0.27)	0.189
**Phosphatidylserine (PS)**	-0.01 (-0.09, 0.06)	0.697	0.00 (-0.06, 0.07)	0.984	0.01 (-0.01, 0.03)	0.539	-0.05 (-0.11, 0.02)	0.255
**Free Cholesterol (COH)**	0.71 (0.54, 0.88)	**<0.001**	0.52 (0.36, 0.68)	**<0.001**	0.07 (0.02, 0.13)	**0.034**	0.22 (0.04, 0.39)	**0.055**
**Cholesterol Ester (CE)**	0.41 (0.26, 0.55)	**<0.001**	0.26 (0.13, 0.40)	**<0.001**	0.02 (-0.03, 0.06)	0.468	0.26 (0.13, 0.39)	**0.001**
**Diacylglycerol (DG)**	0.31 (0.12, 0.50)	**0.002**	0.02 (-0.15, 0.19)	0.820	-0.03 (-0.08, 0.02)	0.318	0.64 (0.52, 0.76)	**<0.001**
**Triacylglycerol (TG)**	0.20 (0.03, 0.38)	**0.030**	-0.02 (-0.18, 0.13)	0.820	-0.04 (-0.09, 0.01)	0.153	0.56 (0.45, 0.67)	**<0.001**

**Abbreviations**: Low-density lipoprotein (**LDL**); High-density lipoprotein (**HDL**)

There were no associations between baseline BMI and lipidome except for two lipid classes, lysoalkylphosphatidylcholine (LPC(O)) and lysophosphatidylethanolamine (LPE) which were significantly negatively associated with baseline BMI following adjustment for age, gender and SBP (β co-efficient -1.07 (95%CI -1.72 to -0.42) p = 0.024 and -1.03 (95%CI -1.67 to -0.39) p = 0.024 respectively).

There was no statistically significant association between baseline plasma lipidome and current CD4+ T cell count or duration of known HIV infection (data not shown). Two lipid species, phosphatidylethanolamine (PE) 40:6 (β coefficient 194313 [95% CI 93138–295487], p = 0.041) and diacyglycerol (DG) 16:0/22:6 (β coefficient 62375. [95% CI 89713–235037], p = 0.008) but no classes were significantly associated with baseline plasma HIV RNA. Nadir CD4+ T cell count was associated with the dihexosylceramide (DHC) class (β coefficient 37.61 [95% CI 16.81–58.42], p = 0.012) only.

### On study change in HIV parameters

The CD4+ T cell count increased from baseline to week 48 (pooled median change 169 cells/μL [IQR 89, 240]) while plasma HIV RNA decreased (pooled median change -68,950 copies/ml [IQR -99950, -13,920]). At week 48 there was no significant difference across treatment arms in CD4 cell count, HIV VL or proportion of participants with a detectable VL (see Table 3). Following 12 weeks of therapy, independent of treatment arm, change in plasma HIV VL was predictive of change in three lipid species; phosphatidylinositol (PI) 34:0 (beta-coefficient 11.66 [95% CI 6.11–17.22], p = 0.008); DG 16:0/20:4 (beta-coefficient 51.32 [95% CI 32.22–70.31], p = 0.0002) and DG 16:0/22:6 (beta-coefficient 25.09 [95% CI 14.91–35.28], p = 0.0007).

**Table 3:** Differences in HIV characteristics and traditional lipid measurements by treatment allocation after 48 weeks of therapy. Median and interquartile range unless otherwise stated. Statistically significant p values highlighted in bold.

Characteristics	Arm 1 Efavirenz (n = 32)	Arm 2 Atazanavir/r (n = 44)	Arm 3 Zidovudine/abacavir (n = 39)	*p-value*^a^			
				Arm 1 vs Arm 2	Arm 2 vs Arm 3	Arm 1 vs Arm 3	Overall^b^
CD4+ T cell count, cells/μL	394 (312–464)	369 (286–503)	360 (249–473)	0.996	0.864	0.930	0.911
Detectable HIV VL,^c^ n (%)	5 (15.6)	5 (11.3)	6 (15.3)	0.818	0.757	0.950	0.824^d^
Total Cholesterol, mmol/l	4.73 (4.19–5.43)	4.26 (3.71–4.75)	3.78 (3.19–4.32)	0.068	**0.024**	**<0.001**	**0.001**
HDL, mmol/l	1.10 (0.95–1.26)	0.96 (0.84–1.13)	0.91 (0.80–1.14)	0.114	0.700	**0.031**	0.103
LDL, mmol/l	2.91 (2.42–3.40)	2.42 (1.91–3.23)	2.20 (1.74–2.72)	0.068	0.553	**0.001**	**0.020**
Triglycerides, mmol/l	1.61 (1.11–2.11)	1.52 (1.07–2.29)	1.11 (0.88–1.61)	1.000	**0.025**	0.088	0.103
Glucose, mmol/l	4.94 (4.70–5.39)	4.94 (4.50–5.21)	4.80 (4.32–5.28)	0.815	0.968	0.546	0.825
Body Mass Index, kg/m^2^	23.99 (21.31–25.96)	24.51 (21.59–26.74)	23.12 (22.08–26.22)	0.908	0.840	0.990	0.911
Systolic Blood Pressure, mmHg	121 (113–131)	120 (110–125)	113 (110–120)	0.557	0.454	0.056	0.148

**Abbreviations:** High Density Lipoprotein Cholesterol (**HDL**); Low Density Lipoprotein Cholesterol (**LDL**); Viral load (**VL**)

^a^ Computed using Mann-Whitney U test, corrected by Dunn-Sidak method

^b^ Computed using the Kruskal-Wallis test, corrected by Benjamini-Hochberg method.

^c^ Detectable defined as >50 copies/ml

^d^ Computed using the chi-square test

### On study change in metabolic measures

Triglyceride levels did not change significantly in the 48 weeks following treatment initiation. HDL cholesterol increased from baseline in all arms with no difference in degree of absolute change between arms (efavirenz 0.18 mmol/L [IQR 0.09, 0.37]; atazanavir/r 0.12 mmol/L [IQR 0.00, 0.22]; zidovudine/abacavir 0.08 mmol/L [IQR -0.05, 0.2];p value = 0.234). Total cholesterol increased in the efavirenz (0.50 mmol/L [IQR 0.25, 0.81] and atazanavir arms (0.48 mmol/L [IQR -0.06, 1.01]) while remaining stable in the zidovudine/abacavir arm (0.10 mmol/L [IQR -0.42, 0.47]), leading to a statistically greater increase in the efavirenz and atazanavir/r arms compared with zidovudine/abacavir (p value = 0.021 and 0.037 respectively). There was a trend towards a greater increase in LDL cholesterol in the efavirenz arm (0.30 mmol/L [IQR -0.20,0.56]) compared with the other treatment regimens which had stable or slight decreases in LDL cholesterol (atazanavir/r 0.16 mmol/L [IQR -0.40,0.89]; zidovudine/abacavir -0.07 [IQR -0.4,0.5]) but this did not reach statistical significance (p value = 0.510). This was reflected in significantly lower total-, and LDL- cholesterol concentrations in the zidovudine/abacavir arm at week 48 (see Table 3).

### Changes in plasma lipidome following initiation of ART

There were a number of significant changes in lipid species and some classes from baseline to week 48 in each of the treatment arms. Two lipid classes, phosphatidylinositol and phosphatidylcholine, increased from baseline in the efavirenz arm while in the atazanavir/r arm two lipid classes, monohexosylceramide and G_M3_ ganglioside, decreased in concentration. No classes changed significantly in the zidovudine/abacavir arm (see Table 4).

**Table 4:** Absolute change in lipid classes from baseline to week 48 in each arm. Median and interquartile ranges (IQR), statistically significant p values highlighted in bold.

Lipid Classes	Arm 1 Efavirenz (n = 32)	*p-value*^*a*^	Arm 2 Atazanavir/r (n = 44)	*p-value*^*a*^	Arm 3 Zidovudine/ abacavir (n = 39)	*p-value*^*a*^
**Cer**	0.065 (-1.01,2.79)	0.489	0.071 (-2.22,1.2)	0.540	-0.022 (-1.61,1.69)	0.978
**MHC**	0.636 (-3.03,2.91)	0.775	-1.52 (-3.02,0.015)	**0.019**	0.001 (-1.51,1.43)	0.720
**DHC**	0.722 (-1.28,2.88)	0.303	0.073 (-1.65,1.59)	1.000	-0.313 (-1.93,1.46)	0.948
**THC**	0.119 (-0.155,0.403)	0.100	-0.086 (-0.311,0.168)	0.480	0.076 (-0.255,0.275)	0.847
**GM3**	0.268 (-0.454,1.41)	0.370	-0.653 (-1.53,0.288)	**0.012**	-0.358 (-0.739,0.506)	1.000
**SM**	31 (-83.6,167)	0.655	1.84 (-157,91)	0.934	25 (-87.3,132)	0.903
**PC**	197 (-46.3,786)	**0.014**	168 (-190,459)	0.294	104 (-58.8,341)	0.087
**PC(O)**	2.66 (-7.97,18)	0.805	1.99 (-23.1,21)	1.000	-1.97 (-13.7,12)	0.989
**PC(P)**	3.3 (-4.06,14)	0.412	3.28 (-4.4,10)	0.305	2.38 (-6.86,12)	0.281
**LPC**	42 (-29.7,188)	0.145	47 (-42,196)	0.065	20 (-50.7,117)	0.264
**LPC(O)**	0.201 (-0.377,0.855)	0.242	0.2 (-0.484,1.02)	0.688	0.132 (-0.218,0.715)	0.345
**PE**	-1.42 (-15.5,14)	0.858	-2.23 (-15.2,8.01)	0.896	-2.43 (-11.2,4.7)	0.990
**PE(O)**	0.461 (-0.408,1.45)	0.466	0.223 (-0.551,1.62)	0.393	0.004 (-1.05,1.23)	0.858
**LPE**	1.26 (-2.86,8.23)	0.452	1.3 (-6.07,9.21)	0.960	1.55 (-4.16,12)	0.141
**PE(P)**	0.305 (-0.958,2.19)	0.443	0.351 (-1.18,1.62)	0.993	0.372 (-1.1,1.81)	0.570
**PI**	10 (-11.6,75)	**0.046**	13 (-17.2,46)	0.429	3.09 (-10.3,26)	0.160
**LPI**	0.34 (-0.972,1.99)	0.953	0.211 (-0.851,1.43)	0.853	0.426 (-0.894,1.49)	0.537
**PS**	0.179 (-4.34,6.92)	0.719	-1.13 (-9.77,2.04)	0.820	0.19 (-2.81,6.12)	0.893
**COH**	168 (-34.5,489)	0.089	132 (-146,378)	0.640	63 (-209,264)	0.421
**CE**	165 (-123,407)	0.183	27 (-327,329)	0.999	66 (-92.5,369)	0.984
**DG**	5.25 (-8.04,31)	0.673	9.06 (-18,22)	0.421	0.749 (-12.2,13)	0.851
**TG**	39 (-98.5,222)	0.701	56 (-182,149)	0.990	25 (-93.7,119)	0.939

**Abbreviations**: Ceramide (**Cer**), Monohexosylceramide (**MHC**), Dihexosylceramide (**DHC**), Trihexosylceramide (**THC**), GM3 ganglioside (**GM3**), Sphingomyelin (**SM**), Phosphatidylcholine (**PC**), Alkylphosphatidylcholine (**PC-O**), Phosphatidylcholine plasmalogen (**PC-P**), Lysophosphatidylcholine (**LPC**), Lysoalkylphosphatidylcholine (**LPC(O)**), Phosphatidylethanolamine (**PE**), Akylphosphatidylethanolamine (**PE-O**), Phosphatidylethanolamine plasmalogen (**PE-P**), Lysophosphatidylethanolamine (**LPE**), Phosphatidylinositol (**PI**), Lysophosphatidylinositol (**LPI**), Phosphatidylserine (**PS**), Free Cholesterol (**COH**), Cholesterol Ester (**Ce**), Diacylglycerol (**DG**), Triacylglycerol (**TG**).

^a^ computed with paired t-test and corrected by Dunn-Sidak method

**Fig 1:**
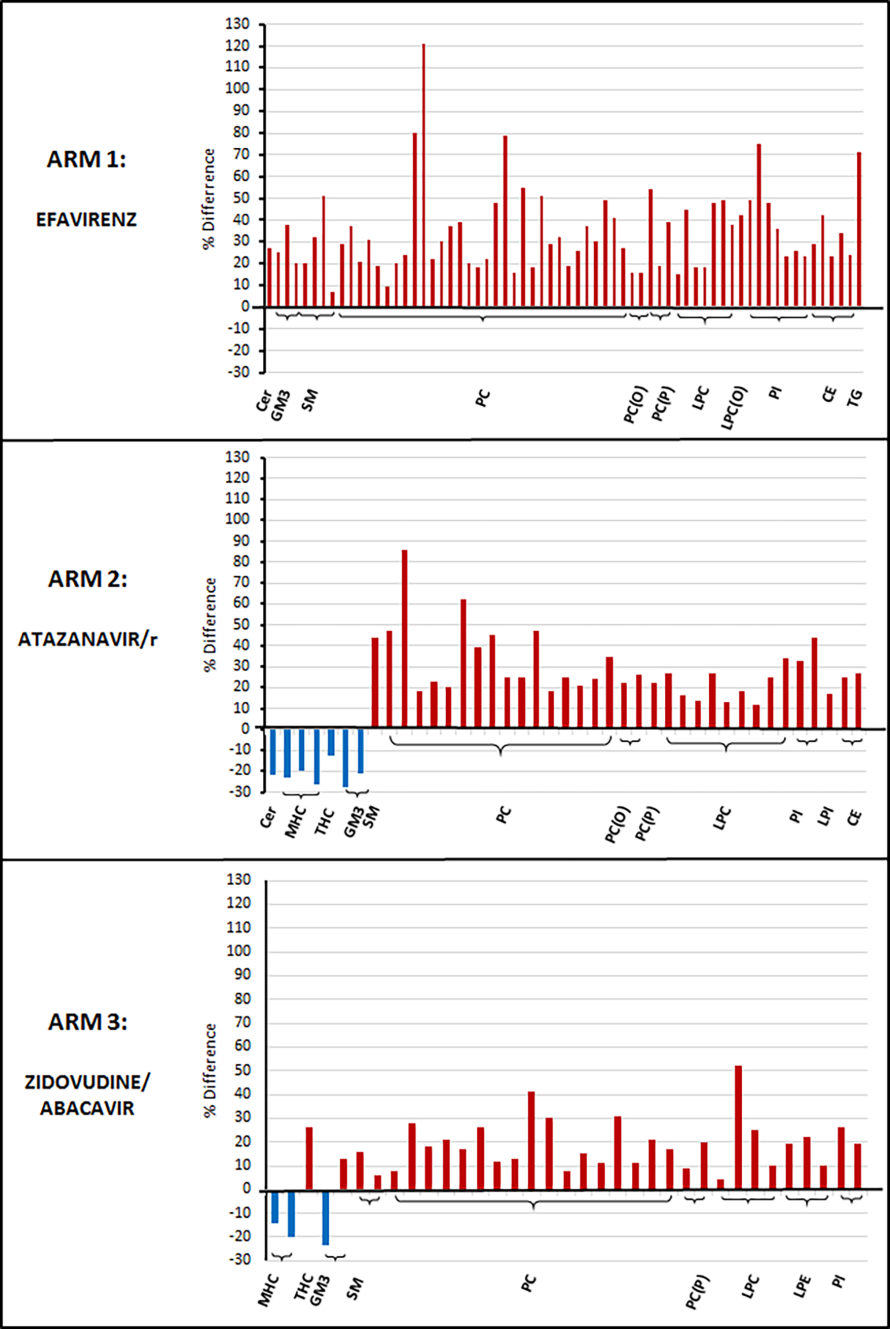
Lipid species which changed significantly from baseline to week 48 by treatment arm. Graph represents the percentage difference from baseline to week 48 in lipid species which changed significantly (defined as p<0.05) by treatment arm, p-values (not shown) computed using paired t-test and corrected by Dunn- Sidak method. **Abbreviations:** Alkylphosphatidylcholine (PC-O), Ceramide (Cer), GM3 ganglioside(GM3), Lysophosphatidylcholine (LPC), Lysophosphatidylinositol (LPI), Lysoalkylphosphatidylcholine (LPC(O)), Monohexosylceramide(MHC), Phosphatidylcholine (PC), Phosphatidylcholine plasmalogen (PC-P), Phosphatidylinositol (PI), Sphingomyelin (SM), Triacylglycerol (TG), Trihexosylceramide (THC).

In addition to the changes observed in lipid classes there were significant changes in a number of lipid species following initiation of ART (see Fig 1). In the efavirenz arm 65 lipid species increased from baseline. In the atazanavir/r arm seven lipid species (from the ceramide, monohexosylceramide, trihexosylceramide and G_M3_ ganglioside classes) decreased in concentration, while a further 34 species increased. In the zidovudine/abacavir arm 31 lipid species increased from baseline and four species (from the monohexosylceramide, G_M3_ ganglioside and phosphatidylcholine classes) decreased (see Fig 1).

**Fig 2:**
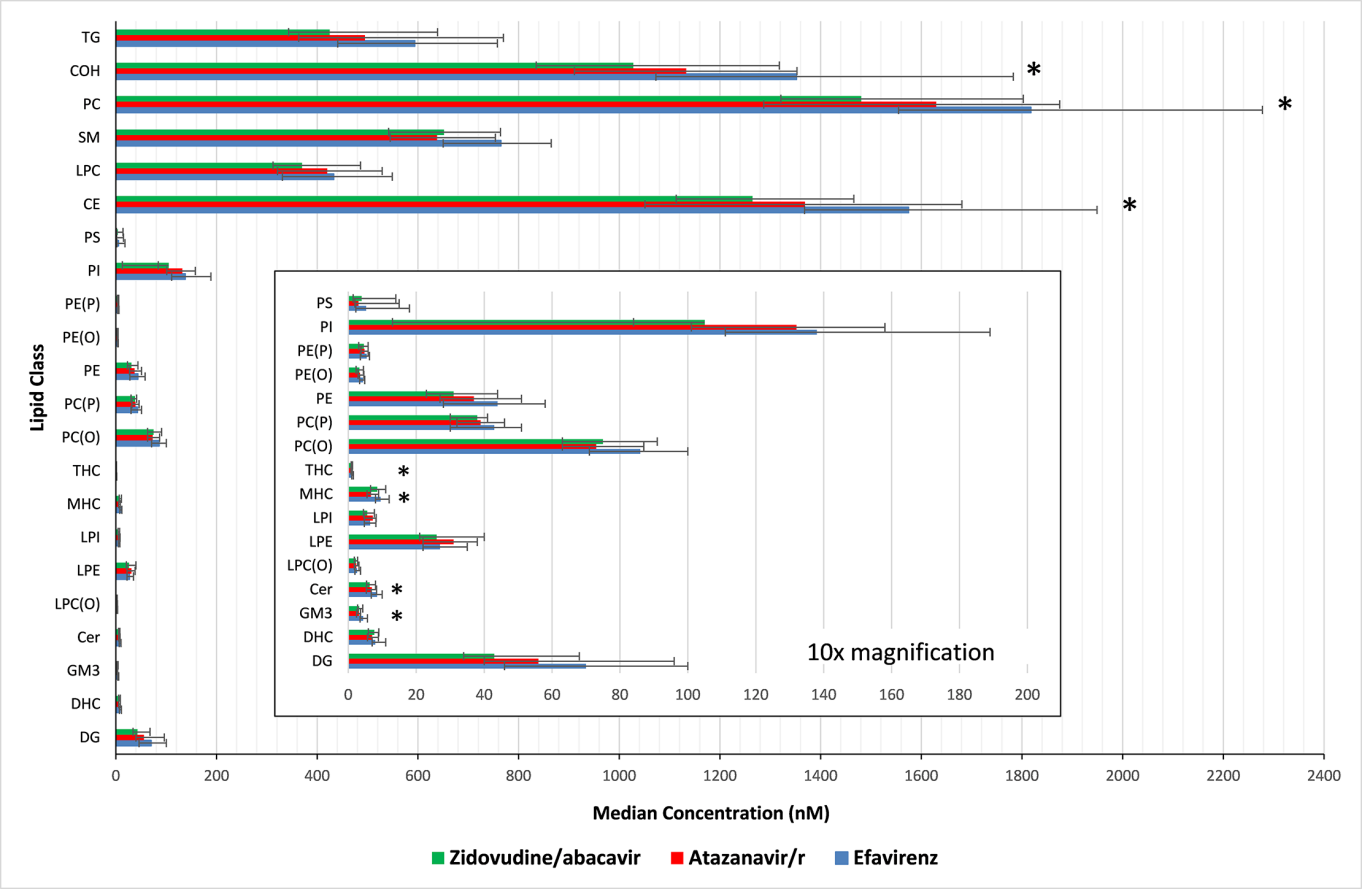
Lipid class concentrations by treatment regimens following 48 weeks of antiretroviral therapy. Insert = 10x magnification of lipid species with lower abundance * p< 0.05 for difference between arms; p-value computed using Kruskal-Wallis test and corrected using Benjamini Hochberg method. **Abbreviations:** Ceramide (**Cer**), Monohexosylceramide (**MHC**), Dihexosylceramide (**DHC**), Trihexosylceramide (**THC**), GM3 ganglioside (**GM3**), Sphingomyelin (**SM**), Phosphatidylcholine (**PC**), Alkylphosphatidylcholine (**PC-O**), Phosphatidylcholine plasmalogen (**PC-P**), Lysophosphatidylcholine (**LPC**), Lysoalkylphosphatidylcholine (**LPC(O)**), Phosphatidylethanolamine (**PE**), Akylphosphatidylethanolamine (**PE-O**), Phosphatidylethanolamine plasmalogen (**PE-P**), Lysophosphatidylethanolamine (**LPE**), Phosphatidylinositol (**PI**), Lysophosphatidylinositol (**LPI**), Phosphatidylserine (**PS**), Free Cholesterol (**COH**), Cholesterol Ester (**Ce**), Diacylglycerol (**DG**), Triacylglycerol (**TG**).

This led to significant differences in 73 lipid species and seven lipid classes by treatment regimen at week 48 (see Fig 2 and S1 Table). Consistently higher lipid concentrations were seen in the efavirenz arm compared with the atazanavir/r or zidovudine/abacavir arms. This was true for all lipid classes with statistically significant differences in concentration at week 48 specifically the; cholesterol ester, free cholesterol, phosphatidylcholine, G_M3_ ganglioside, trihexosylceramide, monohexosylceramide, and ceramide classes.

Neither baseline HIV characteristics (CD4+ T-cell count, nadir CD4+T cell count, HIV VL, duration of HIV infection) nor baseline metabolic results (total cholesterol, LDL, HDL, triglycerides, glucose) predicted the observed change in lipid species at 12 or 48 weeks (data not shown).

### Association between traditional lipid measurements and plasma lipidome at week 48

**Fig 3:**
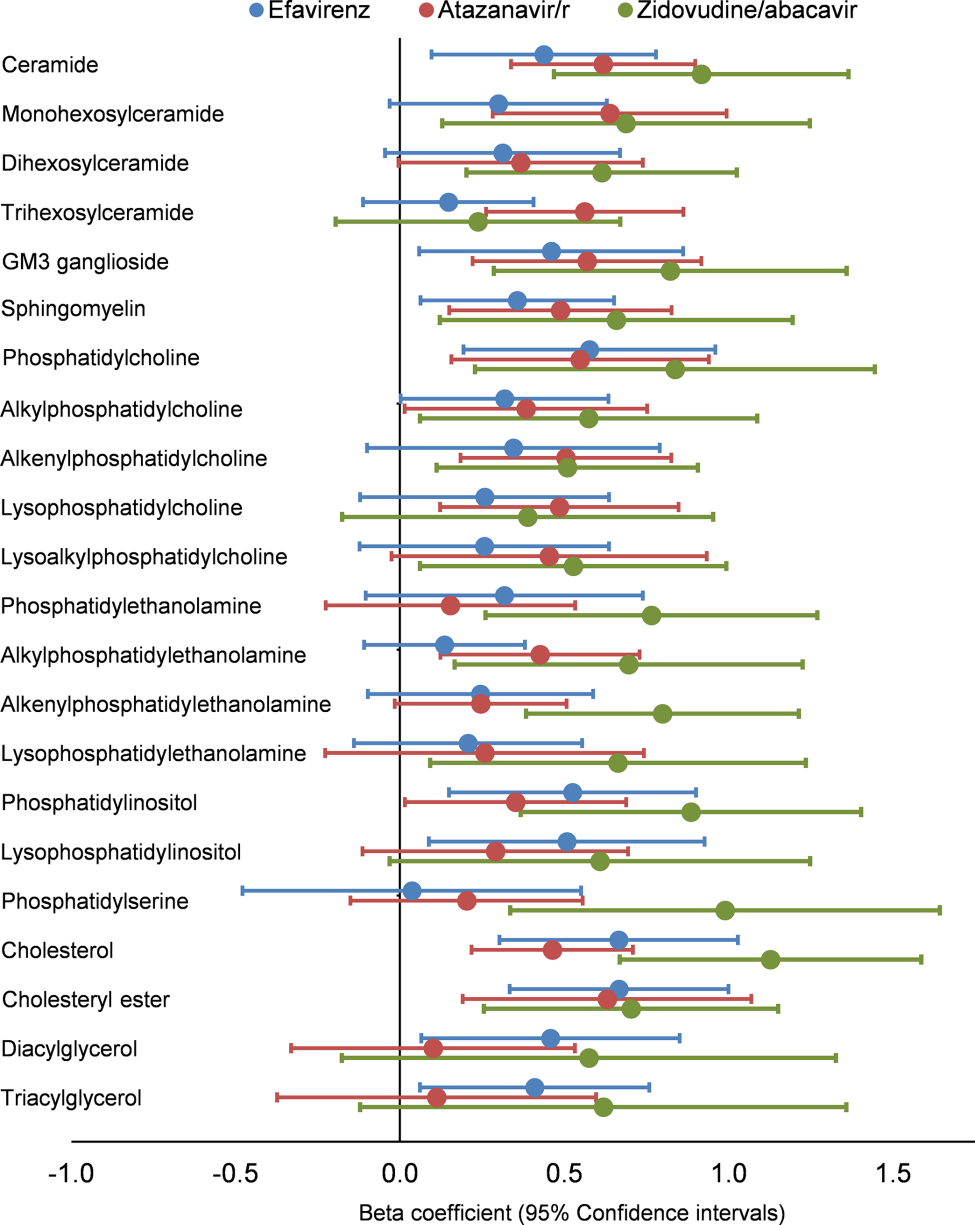
Associations between lipid classes and total cholesterol level following 48 weeks of antiretroviral therapy by treatment allocation. Beta-coefficients represent the change in total cholesterol level associated with each interquartile range increase in lipid class concentration at week 48. Results adjusted for age, gender, body mass index and systolic blood pressure. Lipid classes with statistically significant associations with total cholesterol (defined as p <0.05 following adjustment for multiple comparisons using the Benjamini-Hochberg method) for each arm where—**Efavirenz:** free cholesterol (p = 0.015), cholesterol ester (p = 0.013). **Atazanavir/r:** ceramide (p = 0.002), trihexosylceramide (p = 0.006), free cholesterol (p = 0.006), monohexosylceramide (p = 0.006), GM3 ganglioside (p = 0.012), alkenylphosphatidylcholine (p = 0.014), sphingomyelin (p = 0.021), phosphatidylcholine (p = 0.021), alkylphosphatidylethanolamine (p = 0.021), cholesteryl ester (p = 0.021), lysophosphatidylcholine (p = 0.025). **Zidovudine/abacavir:** ceramide (p = 0.004), monohexosylceramide (p = 0.035), dihexosylceramide (p = 0.015), GM3 ganglioside (p = 0.015), sphingomyelin (p = 0.035), phosphatidylcholine (p = 0.024), alkylphosphatidylcholine (p = 0.046), alkenylphosphatidylcholine (p = 0.031), lysoalkylphosphatidylcholine (p = 0.046), phosphatidylethanolamine (p = 0.015), alkylphosphatidylethanolamine (p = 0.029), alkenylphosphatidylethanolamine (p = 0.005), lysophosphatidylethanolamine (p = 0.043), phosphatidylinositol (p = 0.011), phosphatidylserine (p = 0.015), free cholesterol (p = 0.001), cholesteryl ester (p = 0.015).

There was a strong association between total cholesterol levels with the plasma lipidome at week 48 (17 classes and 111 species significantly associated) in the zidovudine/abacavir arm following adjustment for age, gender, BMI and SBP, as was seen at baseline. In the atazanavir/ ritonavir arm the association between lipidome and total cholesterol was less than observed at baseline but there was still a strong association overall with 11 classes and 105 species significantly associated with week 48 total cholesterol. The efavirenz arm demonstrated the weakest association with only five lipid species and two classes significantly associated with total cholesterol at week 48 (see Fig 3). A similar pattern was seen with LDL-cholesterol with the Zidovudine/abacavir arm demonstrating the strongest correlation between LDL-cholesterol and lipidome and a very weak association seen with the efavirenz and atazanavir/r arms (data not shown).

## Discussion

This study has demonstrated that initiation of ART leads to significant changes in the plasma lipidome and that the individual ARV agents used may impact on specific lipid species as well as the magnitude of the change in lipid species affected. In particular, the efavirenz based regimen demonstrated greater changes in lipidome when compared with a ritonavir-boosted atazanavir or quad-nucleo(t)side combination in this study.

While the investigation of lipidomes in HIV are still in their infancy the effects of individual ARV drugs on traditional measures of cholesterol is well described. In general nucleos(t)ide reverse transcriptase inhibitors (NRTI) are considered essentially lipid neutral [12]. When compared with tenofovir, zidovudine and abacavir are both associated with small increases in total- and LDL- cholesterol although the total- to HDL- cholesterol ratio is, in general, the same across the three agents [13–15]. The degree and pattern of protease inhibitor (PI)- associated dyslipidaemia varies significantly by individual PI agent [16]. Ritonavir has the most deleterious effects on lipid profile while the newer agents (atazanavir and darunavir) have markedly smaller impact on total-, LDL- and HDL- cholesterol [17, 18]. PI therapy leads to decreased clearance of very low density lipoprotein (VLDL) [19], increased hepatic free fatty acid synthesis and decreased uptake of free fatty acids into skeletal muscle and adipose tissue [20, 21]. The elevated circulating levels of free fatty acids associated with PI therapy would be expected to result in elevated de novo synthesis of diacylglycerols and triacylglycerols but this was not observed in this sample set, with the atazanavir/r arm demonstrating similar levels to those in the efavirenz and zidovudine/abacavir arms. This likely reflects the lipid neutral properties of atazanavir compared with other PIs [22]. Further work is needed to determine what effect other PIs (such as darunavir) have on the plasma lipidome.

Greater lipid perturbations have been associated with efavirenz compared with other modern day ARVs, in particular higher total and non-HDL cholesterol [23–25]. This is the result of efavirenz-mediated effects on mitochondria, leading to disruptions in mitochondrial function [26], and reductions in the lipogenic transcription factor sterol regulatory element-binding protein-1c (SREBP-1c) which prevents lipid storage in adipocytes and promotes cholesterol biosynthesis [27].

Given the different mechanisms by which each agent contributes to dyslipidaemia it is reasonable to assume that the lipidome associated with each agent would also be different. Decreases in monohexosylceramide, trihexosylceramide, GM3 ganglioside and ceramides following initiation of atazanavir/r or zidovudine/abacavir compared with increases in the efavirenz arm support this and suggest differential effects on sphingolipid metabolism [28]. While none of the sphingolipids were specifically associated with baseline HIV viral load levels or change in viral load with treatment it is plausible that the effect seen in the atazanavir/r and zidovudine/abacavir arms may be the consequence of HIV suppression rather than a direct effect of the agents themselves on lipid metabolism (i.e. a “return to health” phenomena). Efavirenz-based therapy may be increasing sphingolipids through disturbances in mitochondrial function promoting upregulation of the sphingolipid pathways. However this remains an hypothesis only at this stage and further work is needed to determine the mechanisms by which the different regimens affect lipid metabolism before definitive statements can be made.

The observed increase in sphingolipids may be of clinical importance as they are highly bioactive, contributing to regulation of immune cell migration and enhance neutrophil adhesion to vessel walls [29]. Ceramides are potent regulators of cellular growth and apoptotic cell death, and are important in the development of atherosclerosis through modifications in cardiac contractility, vasomotor responses and endothelial cell function [30]. In coronary arteries, ceramide signalling has been shown to reduce nitric oxide (NO) bioavailability and thus decrease endothelial-dependent coronary vasodilation [31]. Levels of distinct sphingolipids have been shown to have predictive potential for symptomatic CAD that is superior to the currently used LDL measurement, underscoring the value that molecular lipidomic analyses can add to CAD risk stratification [32].

While it has been previously thought that efavirenz induced dyslipidaemia was not atherogenic in nature due to a balanced increase in LDL and HDL (and hence no alteration in LDL/HDL ratio) [24], a recent analysis from the large veterans affairs database in the US has found an increased risk of cardiovascular events with current exposure to efavirenz (OR = 1.40; 95% CI 1.19–1.66) although this has not been seen in other prospective cohort studies [33]. It is not clear if the increase in sphingolipid classes observed in the efavirenz arm (10–35% increase compared with zidovudine/abacavir) is of sufficient magnitude to be clinically relevant and further work is needed to determine this.

Our finding of changes in plasma lipidome with initiation of ART are supported by that of Belury et.al. who demonstrated in a similar demographic of ARV naïve HIV positive individuals that initiation of ART with a raltegravir based regimen led to significant changes in the proportional representation of saturated, monounsaturated and polyunsaturated fatty acids [34]. Of interest they identified increases in a number of lysophosphatidylcholine lipid species (specifically LPC 10:0 and LPC 20:0) with ART initiation which was also seen in all arms of our study, with no difference across arms, suggesting that these changes are not specific to an ARV class and thus may be secondary to changes in HIV driven inflammation and innate immune function.

Since algorithms to predict cardiovascular risk that are useful in the general population often under-call the risk in HIV positive individuals novel CVD risk markers are needed [5]. Lipidomics has excellent potential to expand our diagnostic and treatment capabilities in this area. It has been shown in HIV-negative populations that mass spectrometry-based lipidomic profiling can outperform measurement of conventional lipids as a biomarker of future CVD [35]. The large prospective population-based Bruneck study demonstrated that triacylglycerols (TAGs) and cholesterol esters (CEs) with a low carbon number and double-bond content are strongly associated with future cardiovascular events and addition of lipid species most strongly associated with events to traditional risk factors resulted in improved risk discrimination compared with traditional means alone [36]. Previous work by our group has identified an association between coronary artery disease and increased abundance of specific lipid species [37], and demonstrated that risk prediction models based on lipidomic profiling outperform traditional risk scores in HIV positive individuals [8]. Interestingly of the 74 lipid species found to be associated with future cardiovascular events in HIV positive individuals only 14 (predominantly from the ceramide and phosphatidylcholine classes) were observed to change with initiation of antiretroviral therapy in this current project. Of these, 12 were found in increased abundance in participants on an efavirenz-containing regimen compared with the atazanavir/r or abacavir/zidovudine arms. This supports that the lipidome associated with CVD in people living with HIV is only partially dictated by the choice of ARV regimen with other factors not assessed in this work (such as alterations in gut microbiome and chronic HIV-associated inflammation) likely playing a much greater role. It’s not know whether the increases in these lipids seen with initiation of ART continue to increase with ongoing use past 48 weeks, but if so, this could increase the proportional impact of ART on cardiac risk.

### Limitations

The sample size for this study was reasonably small which may have affected the statistical power. We were unable to adjust for genetic or dietary factors which play an important role in ART-associated dyslipidaemia [38, 39], but given the randomised treatment allocation and similar baseline lipid levels across the arms it is less likely that a significant imbalance in dietary or genetic differences is responsible for our findings. The analysis was performed on unfractionated plasma samples and hence we can only correlate the changes in lipid levels to total LDL and HDL level and cannot differentiate whether the increased in ceramides for instance is within HDL or LDL particles specifically. There were no coronary or cardiovascular events during follow-up (a factor of the young age of participants) and so we are unable to directly correlate the lipid changes seen with individual cardiovascular risk. The results are strengthened however by the international participation in the study which increases applicability to a broad clinical setting. Longer term studies are needed to determine if the observed differences between regimens are maintained and to describe the effects newer ARV agents, such as dolutegravir, and newer PI’s (such as darunavir) and NNRTIs (etravirine and rilpivirine) have on the plasma lipidome.

## Conclusions

Initiation of ART leads to significant changes in plasma lipidome in ARV-naïve HIV positive patients. This effect was most pronounced in those randomised to receive an efavirenz based regimen. Changes in the plasma lipidome may offer additional insights into the effect of ART on lipid classes and species, relevant to cardiovascular risk.

## Supporting information

S1 File Experimental protocol and list of lipid species and classes. (DOCX)

S1 Table Difference in lipid species between arms following 48 weeks of therapy. (DOCX)
