# Supplementary material for: Changes in plasma lipidome following initiation of antiretroviral therapy
Source: PLoS One. 2018 Aug 29;13(8):e0202944. doi: 10.1371/journal.pone.0202944 (PMC6114786; doi:10.1371/journal.pone.0202944)
Supplement: S1 File — (DOCX) [file pone.0202944.s001.docx]

**Supporting Information 1 File**

**Experimental Protocol**

**Sample preparation and lipid extraction.** The plasma samples were randomised for lipid extraction and analysis to minimise experimental bias within the groups. Quality control plasma samples were included at a ratio of 1:10. Total lipid extraction from a 10μL aliquot of plasma was performed by a single phase chloroform:methanol (2:1) extraction [1].

Briefly, a 10µL aliquot of plasma was combined with 200 µL CHCl_3_/MeOH (2:1) and 15µL of internal standard mix. The internal standards comprised lipids which are either stable isotope labelled or non-physiological and so present in plasma at extremely low concentrations. The samples were briefly vortexed, mixed (rotary mixer, 10 min), sonicated (water bath, 30 min) then allowed to stand (20 min) at room temperature. Samples were centrifuged (16,000×g, 10 min) and the supernatant was dried under a stream of nitrogen at 40^o^C. The extracted lipids were resuspended in 50µL H_2_O saturated BuOH with sonication (10 min), followed by 50µL of 10 mM NH_4_COOH in MeOH. Extracts were centrifuged (3,350xg, 5 min) and the supernatant transferred into 0.2 mL glass vials with teflon insert caps.

**High performance liquid chromatography-mass spectrometry analysis.** Lipid analysis was performed by liquid chromatography, electrospray ionisation-tandem mass spectrometry using a Agilent 1200 liquid chromatography system combined with an Applied Biosystems API 4000 Q/TRAP mass spectrometer with a turbo-ionspray source (350^o^C) and Analyst 1.5 data system. Liquid chromatography was performed on a Zorbax C18, 1.8 µm, 50 × 2.1 mm column (Agilent Technologies). Solvents A and B consisted of tetrahydrofuran:methanol:water in the ratio (30:20:50) and (75:20:5) respectively, both containing 10 mM ammonium formate. Columns were heated to 50^o^C and the auto-sampler regulated to 25^o^C. Diacylglycerol and triacylglycerol species (1 µL injection) were separated using an isocratic flow (100 µL/min) of 85% B over 6 minutes. All other lipid species (5µL injection) were separated under gradient conditions (300 µL/min) 0% B to 100% B over 8.0 min, 2.5 min at 100% B, a return to 0% B over 0.5 min then 10.5 min at 0% B prior to the next injection.

We have previously reported the use of precursor ion and neutral loss scans on control plasma extracts to identify the predominant lipid species of the following lipid classes: dihydroceramide (Cer), ceramide (Cer), monohexosylceramide (HexCer), dihexosylceramide (Hex2Cer), trihexosylcermide (Hex3Cer), GM3 ganglioside (GM3), sphingomyelin (SM), phosphatidylcholine (PC), alkylphosphatidylcholine (PC O), alkenylphosphatidylcholine (plasmalogen, PC P), lysophosphatidylcholine (LPC), lysoalkylphosphatidylcholine (lysoplatelet activating factor, LPC O), phosphatidylethanolamine (PE), alkylphosphatidylethanolamine (PE O), alkenylphosphatidylethanolamine (plasmalogen, PE P), lysophosphatidylethanolamine (LPE), phosphatidylinositol (PI), lysophosphatidylinositol (LPI), phosphatidylserine (PS), phosphatidylglycerol (PG), cholesteryl ester (CE), free cholesterol (ST 27:1/OH), diacylglycerol (DG) and triaclyglycerol (TG) [1-4].

Multiple reaction monitoring (MRM) experiments, established for each lipid species, were combined into two scheduled MRM experiments (sMRM) whereby data from each MRM was only collected during its retention time window (± 30sec). Comparative lipid abundances were calculated by relating the peak area of each species to the peak area of the corresponding internal standard. Peak integration was performed using AB Sciex MultiQuant software v1.2. Total measured lipids of each class were calculated by summing the abundance of individual lipid species.

The assignment of isobaric peaks in particular odd-chain diacyl species as well as alkyl and alkenyl species of phosphatidylcholine and phosphatidylethanolamine were made based on product ion scan in both positive and negative mode (to differentiates isobaric species containing acyl linked odd chain fatty acids from those containing alkyl and alkenyl linkages) and relative retention time compared to a series of alkyl and alkenyl standards (Avanti polar lipids and in-house synthesised standards).

The abbreviations shown above are only used when referring to individual lipid species e.g. LPC 22:6 which defines a lysophosphatidylcholine with a fatty acid containing 22 carbons and six double bonds. For a number of the lipids which contain two fatty acid chains the mass spectrometry based measurements here do not directly determine the constituent fatty acids but rather the sum of the number of carbons and the sum of the number of double bonds across both fatty acids. Accordingly, we denote these species as the combined length and number of double bonds, e.g. PC 36:4.

Multiple Reaction Monitoring (MRM) experiments were established for the major species of each lipid class identified in plasma. Although not the major fragmentation pathway, phosphatidylethanolamine plasmalogens were analysed using MRMs based on the neutral loss of 141 as the standard used for these was PE 17:0/17:0 which does have a neutral loss of 141 as the major fragmentation pathway. We recognise that this will affect accuracy of the phosphatidylethanolamine plasmalogen measurements; however, our goal in this study was to achieve precise relative measures of many lipid species and so we have used a common fragmentation for all phosphatidylethanolamine species that are related to the same internal standard.

A total of 331 lipid species distributed across another 24 lipid classes were initially analysed. Relative lipid levels were calculated by relating the peak area of each species to the peak area of the corresponding stable isotope or non-physiological internal standard. Total lipid class levels were calculated as the sum of the individual lipid species levels within each class.

**Assay performance.** Based on the analysis of the 18 quality control samples, 74% of lipid species had a percentage coefficient of variation (%CV) of less than 20% and 93% of lipid species had a %CV less than 30%. Lipid species with a %CV of >30% (n =25) were primarily low abundance plasma species with a high signal-to-noise ratio. These species were excluded from our statistical analysis as their reported measurements were deemed to be unreliable.

**List of lipid classes and species measured**

| **Lipid class** | **Lipid species** |
| --- | --- |
| **Ceramide** | Cer 16:0 |
|  | Cer 20:0 |
|  | Cer 22:0 |
|  | Cer 24:0 |
|  | Cer 24:1 |
| **Monohexosylceramide** | MHC 16:0 |
|  | MHC 18:0 |
|  | MHC 20:0 |
|  | MHC 22:0 |
|  | MHC 24:0 |
|  | MHC 24:1 |
| **Dihexosylceramide** | DHC 16:0 |
|  | DHC 18:0 |
|  | DHC 20:0 |
|  | DHC 22:0 |
|  | DHC 24:0 |
|  | DHC 24:1 |
| **Trihexosylceramide** | THC 16:0 |
|  | THC 20:0 |
|  | THC 22:0 |
|  | THC 24:0 |
|  | THC 24:1 |
| **GM3 ganglioside** | GM3 16:0 |
|  | GM3 18:0 |
|  | GM3 20:0 |
|  | GM3 22:0 |
|  | GM3 24:0 |
|  | GM3 24:1 |
| **Sphingomyelin** | SM 31:1 |
|  | SM 32:0 |
|  | SM 32:1 |
|  | SM 32:2 |
|  | SM 33:1 |
|  | SM 34:0 |
|  | SM 34:1 |
|  | SM 34:2 |
|  | SM 34:3 |
|  | SM 35:1 |
|  | SM 35:2 |
|  | SM 36:1 |
|  | SM 36:2 |
|  | SM 36:3 |
|  | SM 37:2 |
|  | SM 38:1 |
|  | SM 38:2 |
|  | SM 39:1 |
|  | SM 41:1 |
|  | SM 41:2 |
|  | SM 42:1 |
| **Phosphatidylcholine** | PC 28:0 |
|  | PC 29:0 |
|  | PC 30:0 |
|  | PC 31:0 |
|  | PC 31:1 |
|  | PC 32:0 |
|  | PC 32:1 |
|  | PC 32:2 |
|  | PC 32:3 |
|  | PC 33:0 |
|  | PC 33:1 |
|  | PC 33:2 |
|  | PC 33:3 |
|  | PC 34:0 |
|  | PC 34:1 |
|  | PC 34:2 |
|  | PC 34:3 |
|  | PC 34:4 |
|  | PC 34:5 |
|  | PC 35:1 |
|  | PC 35:2 |
|  | PC 35:3 |
|  | PC 35:4 |
|  | PC 36:0 |
|  | PC 36:1 |
|  | PC 36:2 |
|  | PC 36:3 |
|  | PC 36:4a |
|  | PC 36:4b |
|  | PC 36:5 |
|  | PC 36:6 |
|  | PC 37:4 |
|  | PC 37:5 |
|  | PC 37:6 |
|  | PC 38:2 |
|  | PC 38:3 |
|  | PC 38:4 |
|  | PC 38:5 |
|  | PC 38:6a |
|  | PC 38:6b |
|  | PC 38:7 |
|  | PC 39:5 |
|  | PC 39:6 |
|  | PC 39:7 |
|  | PC 40:4 |
|  | PC 40:5 |
|  | PC 40:6 |
|  | PC 40:7 |
|  | PC 40:8 |
| **Alkylphosphatidylcholine** | PC(O-32:0) |
|  | PC(O-32:1) |
|  | PC(O-32:2) |
|  | PC(O-34:1) |
|  | PC(O-34:2) |
|  | PC(O-34:3) |
|  | PC(O-34:4) |
|  | PC(O-35:4) |
|  | PC(O-36:0) |
|  | PC(O-36:1) |
|  | PC(O-36:2) |
|  | PC(O-36:3) |
|  | PC(O-36:4) |
|  | PC(O-36:5) |
|  | PC(O-38:4) |
|  | PC(O-38:5) |
|  | PC(O-40:5) |
|  | PC(O-40:6) |
|  | PC(O-40:7) |
| **Phosphatidylcholine plasmalogen** | PC(P-32:0) |
|  | PC(P-32:1) |
|  | PC(P-34:1) |
|  | PC(P-34:2) |
|  | PC(P-34:3) |
|  | PC(P-36:2) |
|  | PC(P-36:4) |
|  | PC(P-36:5) |
|  | PC(P-38:4) |
|  | PC(P-38:5) |
|  | PC(P-38:6) |
|  | PC(P-40:6) |
| **Lysophosphatidylcholine** | LPC 14:0 |
|  | LPC 15:0 |
|  | LPC 16:0 |
|  | LPC 16:1 |
|  | LPC 17:0 |
|  | LPC 17:1 |
|  | LPC 18:0 |
|  | LPC 18:1 |
|  | LPC 18:2 |
|  | LPC 18:3 |
|  | LPC 20:0 |
|  | LPC 20:1 |
|  | LPC 20:2 |
|  | LPC 20:3 |
|  | LPC 20:4 |
|  | LPC 20:5 |
|  | LPC 22:0 |
|  | LPC 22:1 |
|  | LPC 22:5 |
|  | LPC 22:6 |
|  | LPC 24:0 |
|  | LPC 26:0 |
| **Lysoalkylphosphatidylcholine** | LPC(O-16:0) |
|  | LPC(O-18:0) |
|  | LPC(O-18:1) |
|  | LPC(O-22:0) |
|  | LPC(O-24:0) |
|  | LPC(O-24:1) |
|  | LPC(O-24:2) |
| **Phosphatidylethanolamine** | PE 32:0 |
|  | PE 34:1 |
|  | PE 34:2 |
|  | PE 34:3 |
|  | PE 35:1 |
|  | PE 35:2 |
|  | PE 36:0 |
|  | PE 36:1 |
|  | PE 36:2 |
|  | PE 36:3 |
|  | PE 36:4 |
|  | PE 36:5 |
|  | PE 38:3 |
|  | PE 38:4 |
|  | PE 38:5 |
|  | PE 38:6 |
|  | PE 40:5 |
|  | PE 40:6 |
|  | PE 40:7 |
| **Akylphosphatidylethanolamine** | PE(O-34:1) |
|  | PE(O-34:2) |
|  | PE(O-36:2) |
|  | PE(O-36:3) |
|  | PE(O-36:4) |
|  | PE(O-36:5) |
|  | PE(O-38:4) |
|  | PE(O-38:5) |
|  | PE(O-40:5) |
|  | PE(O-40:6) |
| **Phosphatidylethanolamine plasmalogen** | PE(P-34:1) |
|  | PE(P-34:2) |
|  | PE(P-36:1) |
|  | PE(P-36:2) |
|  | PE(P-36:4) |
|  | PE(P-38:4) |
|  | PE(P-38:5) |
|  | PE(P-38:6) |
|  | PE(P-40:5) |
|  | PE(P-40:6) |
| **Lysophosphatidylethanolamine** | LPE 16:0 |
|  | LPE 18:0 |
|  | LPE 18:1 |
|  | LPE 18:2 |
|  | LPE 20:4 |
|  | LPE 22:6 |
| **Phosphatidylinositol** | PI 32:0 |
|  | PI 32:1 |
|  | PI 34:0 |
|  | PI 34:1 |
|  | PI 36:1 |
|  | PI 36:2 |
|  | PI 36:3 |
|  | PI 36:4 |
|  | PI 38:2 |
|  | PI 38:3 |
|  | PI 38:4 |
|  | PI 38:5 |
|  | PI 38:6 |
|  | PI 40:4 |
|  | PI 40:5 |
|  | PI 40:6 |
| **Lysophosphatidylinositol** | LPI 18:0 |
|  | LPI 18:1 |
|  | LPI 18:2 |
|  | LPI 20:4 |
| **Phosphatidylserine** | PS 36:1 |
|  | PS 38:4 |
|  | PS 40:6 |
| **Free Cholesterol** | COH |
| **Cholesterol Ester** | CE 14:0 |
|  | CE 15:0 |
|  | CE 16:0 |
|  | CE 16:1 |
|  | CE 16:2 |
|  | CE 17:0 |
|  | CE 17:1 |
|  | CE 18:0 |
|  | CE 18:1 |
|  | CE 18:2 |
|  | CE 18:3 |
|  | CE 20:1 |
|  | CE 20:2 |
|  | CE 20:3 |
|  | CE 20:4 |
|  | CE 20:5 |
|  | CE 22:0 |
|  | CE 22:1 |
|  | CE 22:4 |
|  | CE 22:5 |
|  | CE 22:6 |
|  | CE 24:1 |
|  | CE 24:5 |
| **Diacylglycerol** | DG 14:0/18:1 |
|  | DG 14:0/18:2 |
|  | DG 16:0/18:1 |
|  | DG 16:0/20:3 |
|  | DG 16:0/20:4 |
|  | DG 16:0/22:5 |
|  | DG 16:0/22:6 |
|  | DG 18:0/18:2 |
|  | DG 18:0/20:4 |
|  | DG 18:1/18:1 |
|  | DG 18:1/18:2 |
|  | DG 18:1/18:3 |
|  | DG 18:1/20:3 |
|  | DG 18:1/20:4 |
|  | DG 18:2/18:2 |
| **Triacylglycerol** | TG 14:0/16:0/18:1 |
|  | TG 14:0/16:0/18:2 |
|  | TG 14:0/16:1/18:1 |
|  | TG 14:0/16:1/18:2 |
|  | TG 14:0/17:0/18:1 |
|  | TG 14:0/18:0/18:1 |
|  | TG 14:0/18:2/18:2 |
|  | TG 14:1/16:0/18:1 |
|  | TG 14:1/18:0/18:2 |
|  | TG 14:1/18:1/18:1 |
|  | TG 15:0/16:0/18:1 |
|  | TG 15:0/18:1/18:1 |
|  | TG 16:0/16:0/18:0 |
|  | TG 16:0/16:0/18:1 |
|  | TG 16:0/16:0/18:2 |
|  | TG 16:0/16:1/17:0 |
|  | TG 16:0/16:1/18:1 |
|  | TG 16:0/17:0/18:0 |
|  | TG 16:0/17:0/18:1 |
|  | TG 16:0/17:0/18:2 |
|  | TG 16:0/18:0/18:1 |
|  | TG 16:0/18:1/18:1 |
|  | TG 16:0/18:1/18:2 |
|  | TG 16:0/18:2/18:2 |
|  | TG 16:1/16:1/16:1 |
|  | TG 16:1/16:1/18:0 |
|  | TG 16:1/16:1/18:1 |
|  | TG 16:1/17:0/18:1 |
|  | TG 16:1/18:1/18:1 |
|  | TG 16:1/18:1/18:2 |
|  | TG 17:0/18:1/18:1 |
|  | TG 18:0/18:0/18:1 |
|  | TG 18:0/18:1/18:1 |
|  | TG 18:0/18:2/18:2 |
|  | TG 18:1/18:1/18:1 |
|  | TG 18:1/18:1/18:2 |
|  | TG 18:1/18:1/20:4 |
|  | TG 18:1/18:1/22:6 |
|  | TG 18:1/18:2/18:2 |
|  | TG 18:2/18:2/18:2 |
|  | TG 18:2/18:2/20:4 |

**References**

1. Weir JM, Wong G, Barlow CK, Greeve MA, Kowalczyk A, Almasy L*, et al.* Plasma lipid profiling in a large population-based cohort. *J Lipid Res* 2013,**54**:2898-2908.

2. Meikle PJ, Wong G, Tsorotes D, Barlow CK, Weir JM, Christopher MJ*, et al.* Plasma lipidomic analysis of stable and unstable coronary artery disease. *Arterioscler Thromb Vasc Biol* 2011,**31**:2723-2732.

3. Borg ML, Andrews ZB, Duh EJ, Zechner R, Meikle PJ, Watt MJ. Pigment epithelium-derived factor regulates lipid metabolism via adipose triglyceride lipase. *Diabetes* 2011,**60**:1458-1466.

4. Boslem E, MacIntosh G, Preston AM, Bartley C, Busch AK, Fuller M*, et al.* A lipidomic screen of palmitate-treated MIN6 beta-cells links sphingolipid metabolites with endoplasmic reticulum (ER) stress and impaired protein trafficking. *Biochem J* 2011,**435**:267-276.
