# Supplementary material for: Changes in plasma lipidome following initiation of antiretroviral therapy
Source: PLoS One. 2018 Aug 29;13(8):e0202944. doi: 10.1371/journal.pone.0202944 (PMC6114786; doi:10.1371/journal.pone.0202944)
Supplement: S1 Table — (DOCX) [file pone.0202944.s002.docx]

S1 Table: Difference in lipid species between arms following 48 weeks of therapy

| **Lipid Species** | **Arm 1**  **Efavirenz**  (n = 32) | **Arm 2**  **Atazanavir/r**  (n = 44) | **Arm 3**  **Zidovudine/abacavir**  (n = 39) | *p*-value^a^  (Arm 1 vs Arm 2) | *p*-value^a^  (Arm 2 vs Arm 3) | *p*-value^a^  (Arm 1 vs Arm 3) | *p*-value^b^ |
| --- | --- | --- | --- | --- | --- | --- | --- |
| Cer 16:0 | 0.612 (0.5,0.784) | 0.434 (0.343,0.531) | 0.492 (0.405,0.592) | **<0.0001** | 0.333 | **0.024** | **0.005** |
| Cer 20:0 | 0.246 (0.184,0.374) | 0.163 (0.115,0.208) | 0.146 (0.126,0.225) | **<0.001** | 0.986 | **<0.001** | **0.005** |
| Cer 22:0 | 1.39 (0.942,1.76) | 1.08 (0.871,1.31) | 0.959 (0.836,1.31) | 0.067 | 0.942 | **0.029** | 0.07 |
| Cer 24:0 | 4.40 (3.59,5.74) | 3.92 (2.89,4.80) | 3.40 (2.79,4.74) | 0.194 | 0.913 | **0.046** | 0.127 |
| Cer 24:1 | 1.64 (1.35,2.04) | 1.26 (0.9,1.75) | 1.28 (1.09,1.57) | **0.001** | 0.974 | **0.001** | **0.007** |
| MHC 16:0 | 1.40 (1.12,1.99) | 1.07 (0.887,1.40) | 1.34 (1.05,1.55) | **0.004** | 0.146 | 0.358 | **0.03** |
| MHC 18:0 | 0.211 (0.163,0.255) | 0.141 (0.109,0.178) | 0.177 (0.15,0.203) | **<0.001** | **0.046** | 0.11 | **0.005** |
| MHC 20:0 | 0.227 (0.186,0.282) | 0.161 (0.124,0.221) | 0.188 (0.158,0.261) | **<0.001** | 0.127 | 0.197 | **0.013** |
| MHC 22:0 | 2.04 (1.54,2.78) | 1.60 (1.20,2.26) | 1.80 (1.47,2.31) | **0.012** | 0.235 | 0.523 | 0.055 |
| MHC 24:0 | 3.18 (2.57,4.49) | 2.38 (1.87,3.41) | 2.83 (2.29,3.69) | **0.006** | 0.284 | 0.27 | **0.037** |
| MHC 24:1 | 2.24 (1.79,2.88) | 1.43 (1.20,1.86) | 1.89 (1.42,2.33) | **<0.001** | 0.141 | 0.154 | **0.013** |
| DHC 16:0 | 5.38 (4.66,6.67) | 4.61 (3.98,5.68) | 4.79 (3.72,5.86) | 0.163 | 0.999 | 0.325 | 0.262 |
| DHC 18:0 | 0.126 (0.0895,0.159) | 0.101 (0.0698,0.124) | 0.095 (0.0808,0.123) | **0.027** | 0.872 | 0.107 | 0.074 |
| DHC 20:0 | 0.141 (0.0817,0.175) | 0.0964 (0.0647,0.128) | 0.0927 (0.0646,0.137) | 0.08 | 1 | 0.183 | 0.157 |
| DHC 22:0 | 0.554 (0.334,0.704) | 0.375 (0.267,0.56) | 0.344 (0.248,0.649) | 0.167 | 0.976 | 0.248 | 0.238 |
| DHC 24:0 | 0.517 (0.318,0.674) | 0.356 (0.273,0.525) | 0.396 (0.267,0.582) | 0.076 | 0.936 | 0.338 | 0.183 |
| DHC 24:1 | 1.28 (1.15,1.89) | 1.07 (0.898,1.66) | 1.27 (0.961,1.73) | 0.051 | 0.62 | 0.793 | 0.179 |
| THC 16:0 | 0.0836 (0.0659,0.0942) | 0.0658 (0.0521,0.0788) | 0.0928 (0.0755,0.113) | **0.008** | **<0.0001** | 0.192 | **0.001** |
| THC 20:0 | 0.0637 (0.0484,0.073) | 0.0421 (0.0302,0.0608) | 0.0464 (0.0314,0.0652) | **0.001** | 0.881 | **0.021** | **0.019** |
| THC 22:0 | 0.239 (0.215,0.36) | 0.195 (0.156,0.263) | 0.201 (0.171,0.308) | **0.039** | 0.804 | 0.17 | 0.102 |
| THC 24:0 | 0.312 (0.244,0.394) | 0.25 (0.195,0.303) | 0.251 (0.184,0.318) | **0.007** | 0.996 | **0.04** | **0.037** |
| THC 24:1 | 0.537 (0.44,0.642) | 0.401 (0.315,0.498) | 0.465 (0.352,0.598) | **<0.001** | 0.159 | 0.104 | **0.008** |
| GM3 16:0 | 0.984 (0.853,1.28) | 0.772 (0.611,0.995) | 0.888 (0.766,1.11) | **0.001** | 0.073 | 0.386 | **0.018** |
| GM3 18:0 | 0.539 (0.438,0.633) | 0.372 (0.275,0.473) | 0.398 (0.341,0.497) | **<0.001** | 0.399 | **0.007** | **0.005** |
| GM3 20:0 | 0.293 (0.24,0.422) | 0.205 (0.153,0.261) | 0.216 (0.171,0.284) | **<0.0001** | 0.469 | **0.001** | **0.001** |
| GM3 22:0 | 0.806 (0.611,1.18) | 0.55 (0.447,0.711) | 0.575 (0.451,0.744) | **0.001** | 0.85 | **0.014** | **0.013** |
| GM3 24:0 | 0.645 (0.531,0.869) | 0.459 (0.361,0.57) | 0.49 (0.368,0.628) | **<0.001** | 0.676 | **0.009** | **0.006** |
| GM3 24:1 | 0.973 (0.788,1.34) | 0.616 (0.483,0.83) | 0.749 (0.575,1.05) | **<0.0001** | 0.175 | **0.013** | **0.001** |
| SM 31:1 | 0.378 (0.25,0.492) | 0.288 (0.223,0.465) | 0.296 (0.225,0.376) | 0.635 | 0.85 | 0.188 | 0.322 |
| SM 32:0 | 0.503 (0.414,0.756) | 0.507 (0.359,0.645) | 0.476 (0.354,0.593) | 0.686 | 0.85 | 0.415 | 0.429 |
| SM 32:1 | 17 (14,23) | 16 (12,19) | 14 (11,18) | 0.421 | 0.601 | 0.07 | 0.157 |
| SM 32:2 | 0.971 (0.817,1.35) | 0.821 (0.629,1.07) | 0.765 (0.61,1.03) | 0.129 | 0.942 | **0.044** | 0.11 |
| SM 33:1 | 11 (5.96,12) | 7.47 (6.03,12) | 8.10 (5.98,11) | 0.462 | 0.974 | 0.57 | 0.447 |
| SM 34:0 | 17 (14,21) | 13 (11,17) | 14 (12,17) | **0.003** | 0.868 | **0.03** | **0.025** |
| SM 34:1 | 221 (189,266) | 177 (141,223) | 190 (164,227) | **0.001** | 0.639 | **0.046** | **0.019** |
| SM 34:2 | 28 (23,38) | 23 (19,29) | 25 (20,30) | **0.016** | 0.854 | 0.116 | 0.063 |
| SM 34:3 | 0.261 (0.181,0.337) | 0.22 (0.153,0.314) | 0.218 (0.147,0.315) | 0.469 | 0.982 | 0.288 | 0.362 |
| SM 35:1 | 6.91 (4.77,8.75) | 5.23 (3.78,7.53) | 5.84 (4.25,7.75) | 0.24 | 0.926 | 0.554 | 0.339 |
| SM 35:2 | 1.12 (0.795,1.46) | 0.831 (0.565,1.16) | 0.873 (0.636,1.27) | 0.152 | 0.825 | 0.43 | 0.248 |
| SM 36:1 | 44 (36,52) | 30 (25,41) | 33 (27,41) | **<0.001** | 0.815 | **0.003** | **0.006** |
| SM 36:2 | 18 (13,23) | 12 (9.45,16) | 13 (10,16) | **<0.001** | 0.556 | **0.007** | **0.006** |
| SM 36:3 | 1.32 (1.00,2.09) | 1.13 (0.831,1.66) | 1.24 (0.847,1.68) | 0.455 | 0.969 | 0.833 | 0.515 |
| SM 37:2 | 5.77 (3.91,7.71) | 5.58 (4.71,6.55) | 4.85 (3.74,6.83) | 0.914 | 0.62 | 0.53 | 0.465 |
| SM 38:1 | 320 (252,379) | 289 (244,350) | 300 (249,372) | 0.686 | 0.979 | 0.919 | 0.669 |
| SM 38:2 | 15 (8.63,18) | 12 (9.01,16) | 10 (8.77,14) | 0.664 | 0.488 | 0.162 | 0.246 |
| SM 39:1 | 13 (8.89,16) | 11 (6.89,15) | 9.63 (7.26,15) | 0.992 | 0.942 | 0.758 | 0.746 |
| SM 41:1 | 3.72 (2.93,4.90) | 3.04 (2.31,4.04) | 3.21 (2.47,4.15) | 0.058 | 0.939 | 0.15 | 0.126 |
| SM 41:2 | 3.28 (2.00,4.79) | 2.73 (1.58,4.35) | 1.98 (1.38,2.89) | 0.714 | 0.117 | **0.007** | **0.041** |
| SM 42:1 | 19 (16,24) | 15 (11,18) | 14 (11,17) | **0.003** | 0.994 | **0.001** | **0.011** |
| PC 28:0 | 0.19 (0.106,0.405) | 0.208 (0.116,0.281) | 0.148 (0.0761,0.245) | 0.868 | 0.308 | 0.154 | 0.22 |
| PC 29:0 | 0.171 (0.143,0.225) | 0.139 (0.108,0.175) | 0.143 (0.107,0.198) | **0.018** | 0.999 | 0.104 | 0.071 |
| PC 30:0 | 3.39 (2.25,4.49) | 3.12 (1.90,4.28) | 2.57 (1.84,4.04) | 0.628 | 0.939 | 0.325 | 0.403 |
| PC 31:0 | 0.996 (0.688,1.36) | 0.861 (0.558,1.25) | 0.862 (0.659,1.24) | 0.841 | 0.999 | 0.939 | 0.795 |
| PC 31:1 | 1.53 (1.05,1.89) | 1.21 (0.868,1.63) | 1.21 (0.939,1.77) | 0.286 | 0.93 | 0.523 | 0.357 |
| PC 32:0 | 17 (15,21) | 13 (12,17) | 16 (13,19) | **0.016** | 0.146 | 0.658 | 0.055 |
| PC 32:1 | 23 (17,31) | 20 (14,26) | 18 (15,26) | 0.32 | 0.997 | 0.237 | 0.304 |
| PC 32:2 | 6.51 (5.13,8.83) | 5.64 (4.48,7.13) | 5.18 (4.23,7.09) | 0.216 | 0.905 | 0.076 | 0.156 |
| PC 32:3 | 0.374 (0.278,0.531) | 0.302 (0.244,0.396) | 0.279 (0.227,0.414) | 0.394 | 0.901 | 0.253 | 0.311 |
| PC 33:0 | 1.76 (1.39,2.20) | 1.51 (1.09,2.00) | 1.53 (1.31,2.04) | 0.363 | 0.901 | 0.611 | 0.403 |
| PC 33:1 | 4.80 (4.14,6.53) | 4.86 (3.64,6.15) | 4.83 (3.41,6.35) | 0.793 | 0.995 | 0.936 | 0.763 |
| PC 33:2 | 3.31 (2.53,4.64) | 3.23 (2.26,4.25) | 2.97 (2.40,4.03) | 0.993 | 1 | 0.874 | 0.877 |
| PC 33:3 | 0.0601 (0.0399,0.0808) | 0.046 (0.0305,0.0777) | 0.0438 (0.0271,0.0584) | 0.868 | 0.695 | 0.158 | 0.32 |
| PC 34:0 | 5.51 (4.18,6.57) | 3.85 (3.36,5.71) | 4.07 (3.08,5.36) | **0.022** | 0.965 | **0.015** | **0.037** |
| PC 34:1 | 235 (202,298) | 209 (163,252) | 209 (181,251) | 0.085 | 0.936 | 0.197 | 0.156 |
| PC 34:2 | 436 (343,513) | 387 (324,442) | 403 (328,485) | 0.621 | 0.947 | 0.869 | 0.593 |
| PC 34:3 | 21 (12,24) | 16 (11,21) | 14 (10,18) | 0.768 | 0.399 | 0.154 | 0.233 |
| PC 34:4 | 1.86 (1.30,2.43) | 1.79 (1.07,2.27) | 1.16 (0.906,1.70) | 0.863 | 0.059 | **0.014** | **0.041** |
| PC 34:5 | 0.157 (0.078,0.269) | 0.0921 (0.0559,0.159) | 0.0611 (0.0348,0.0922) | 0.235 | **0.021** | **<0.001** | **0.006** |
| PC 35:1 | 8.44 (7.40,11) | 7.98 (5.72,11) | 7.78 (6.13,10) | 0.635 | 0.998 | 0.325 | 0.429 |
| PC 35:2 | 14 (11,17) | 14 (9.19,17) | 12 (9.18,18) | 0.941 | 0.999 | 0.845 | 0.801 |
| PC 35:3 | 2.34 (1.52,2.81) | 2.16 (1.40,2.88) | 1.69 (1.47,2.44) | 0.998 | 0.422 | 0.264 | 0.333 |
| PC 35:4 | 1.33 (0.955,1.74) | 1.23 (0.776,1.67) | 1.05 (0.653,1.36) | 0.781 | 0.835 | 0.319 | 0.406 |
| PC 36:0 | 0.55 (0.372,0.714) | 0.374 (0.271,0.57) | 0.346 (0.254,0.59) | 0.06 | 0.689 | **0.014** | **0.041** |
| PC 36:1 | 86 (66,112) | 61 (49,89) | 57 (43,78) | **0.007** | 0.469 | **<0.001** | **0.007** |
| PC 36:2 | 18 (14,21) | 16 (12,19) | 13 (11,16) | 0.533 | 0.064 | **0.003** | **0.024** |
| PC 36:3 | 200 (165,245) | 178 (137,210) | 150 (131,183) | 0.356 | 0.1 | **0.002** | **0.022** |
| PC 36:4a | 17 (12,28) | 17 (11,25) | 14 (8.90,19) | 0.953 | 0.298 | 0.27 | 0.284 |
| PC 36:4b | 169 (132,196) | 154 (114,186) | 153 (121,177) | 0.226 | 1 | 0.221 | 0.258 |
| PC 36:5 | 23 (14,37) | 15 (9.20,25) | 13 (8.05,19) | 0.174 | 0.439 | **0.005** | **0.038** |
| PC 36:6 | 0.729 (0.443,1.25) | 0.717 (0.473,1.04) | 0.448 (0.296,0.689) | 0.914 | **0.013** | **0.015** | **0.028** |
| PC 37:4 | 8.48 (6.50,10) | 7.51 (5.17,9.28) | 6.95 (5.78,9.21) | 0.326 | 0.981 | 0.211 | 0.287 |
| PC 37:5 | 1.54 (0.918,1.82) | 0.962 (0.631,1.58) | 0.916 (0.631,1.25) | 0.145 | 0.766 | **0.003** | **0.039** |
| PC 37:6 | 0.525 (0.397,0.872) | 0.523 (0.334,0.778) | 0.464 (0.326,0.607) | 0.969 | 0.777 | 0.408 | 0.487 |
| PC 38:2 | 11 (8.41,14) | 8.37 (6.57,10) | 7.61 (5.66,10) | **0.006** | 0.953 | **0.005** | **0.019** |
| PC 38:3 | 91 (65,115) | 67 (47,92) | 42 (37,63) | 0.074 | **0.01** | **<0.0001** | **0.001** |
| PC 38:4 | 172 (120,203) | 126 (99,157) | 119 (104,155) | **0.033** | 0.986 | **0.006** | **0.035** |
| PC 38:5 | 91 (69,108) | 71 (50,94) | 65 (54,75) | **0.044** | 0.777 | **<0.001** | **0.019** |
| PC 38:6a | 5.99 (4.16,9.36) | 6.29 (4.52,8.26) | 4.84 (3.64,6.52) | 0.888 | 0.146 | 0.104 | 0.141 |
| PC 38:6b | 72 (49,116) | 63 (50,79) | 61 (43,81) | 0.408 | 0.923 | 0.253 | 0.318 |
| PC 38:7 | 1.94 (1.18,2.54) | 1.76 (1.08,2.31) | 1.19 (0.848,1.77) | 0.65 | 0.07 | **0.004** | **0.03** |
| PC 39:5 | 1.79 (1.32,2.22) | 1.34 (1.00,1.82) | 1.19 (0.911,1.50) | 0.058 | 0.743 | **0.003** | **0.03** |
| PC 39:6 | 2.28 (1.57,3.27) | 2.00 (1.36,2.76) | 1.80 (1.35,2.36) | 0.512 | 0.845 | 0.11 | 0.246 |
| PC 39:7 | 0.0852 (0.0733,0.0945) | 0.0728 (0.0503,0.0898) | 0.0693 (0.0381,0.0973) | 0.149 | 0.749 | 0.197 | 0.183 |
| PC 40:4 | 7.36 (4.91,9.97) | 4.76 (3.34,6.79) | 4.11 (3.43,6.75) | **0.024** | 0.992 | **0.007** | **0.034** |
| PC 40:5 | 29 (20,45) | 20 (14,27) | 17 (15,22) | **0.002** | 0.825 | **<0.0001** | **0.003** |
| PC 40:6 | 34 (21,60) | 27 (20,40) | 25 (17,32) | 0.152 | 0.81 | **0.035** | 0.1 |
| PC 40:7 | 7.08 (6.08,9.23) | 6.62 (4.43,9.41) | 5.45 (4.12,7.55) | 0.657 | 0.404 | **0.019** | 0.102 |
| PC 40:8 | 1.55 (1.13,2.19) | 1.53 (0.973,2.13) | 1.23 (0.983,1.69) | 0.748 | 0.664 | 0.226 | 0.322 |
| PC(O-32:0) | 2.71 (2.41,3.29) | 2.18 (1.78,2.84) | 2.41 (1.96,3.20) | **0.004** | 0.313 | 0.288 | **0.035** |
| PC(O-32:1) | 0.573 (0.509,0.693) | 0.463 (0.404,0.573) | 0.508 (0.391,0.641) | **0.009** | 0.92 | 0.126 | 0.055 |
| PC(O-32:2) | 0.0992 (0.0709,0.145) | 0.0839 (0.0551,0.134) | 0.0776 (0.0488,0.126) | 0.57 | 0.958 | 0.358 | 0.403 |
| PC(O-34:1) | 5.39 (4.87,6.53) | 4.36 (3.80,5.33) | 5.34 (4.06,6.09) | **0.001** | 0.144 | 0.546 | **0.022** |
| PC(O-34:2) | 5.39 (4.26,7.83) | 5.24 (4.29,6.36) | 4.97 (3.29,6.81) | 0.793 | 0.766 | 0.338 | 0.403 |
| PC(O-34:3) | 0.168 (0.109,0.256) | 0.155 (0.104,0.222) | 0.175 (0.0908,0.237) | 0.926 | 0.994 | 0.97 | 0.866 |
| PC(O-34:4) | 0.276 (0.184,0.386) | 0.233 (0.193,0.342) | 0.218 (0.158,0.346) | 0.781 | 0.905 | 0.619 | 0.549 |
| PC(O-35:4) | 0.224 (0.144,0.316) | 0.221 (0.167,0.31) | 0.217 (0.146,0.329) | 1 | 0.999 | 1 | 0.998 |
| PC(O-36:0) | 0.0599 (0.0438,0.121) | 0.0444 (0.0313,0.0691) | 0.0487 (0.034,0.0922) | 0.126 | 0.771 | 0.643 | 0.258 |
| PC(O-36:1) | 0.4 (0.337,0.542) | 0.299 (0.248,0.445) | 0.337 (0.27,0.481) | 0.069 | 0.743 | 0.358 | 0.156 |
| PC(O-36:2) | 3.39 (2.58,5.36) | 3.08 (2.46,3.80) | 2.88 (2.35,4.20) | 0.483 | 0.992 | 0.515 | 0.447 |
| PC(O-36:3) | 7.44 (6.00,8.67) | 6.41 (5.17,7.65) | 5.86 (4.71,7.32) | 0.142 | 0.794 | 0.076 | 0.127 |
| PC(O-36:4) | 19 (15,23) | 17 (14,21) | 16 (13,20) | 0.26 | 0.996 | 0.312 | 0.31 |
| PC(O-36:5) | 0.88 (0.55,1.60) | 0.592 (0.459,1.15) | 0.652 (0.419,0.943) | 0.23 | 0.971 | 0.136 | 0.211 |
| PC(O-38:4) | 13 (10,17) | 11 (10,14) | 12 (9.72,13) | 0.519 | 0.994 | 0.728 | 0.523 |
| PC(O-38:5) | 17 (14,20) | 15 (13,18) | 16 (13,18) | 0.314 | 0.707 | 0.863 | 0.394 |
| PC(O-40:5) | 3.81 (3.09,4.53) | 3.13 (2.66,3.78) | 3.21 (2.65,3.80) | 0.052 | 0.923 | 0.154 | 0.121 |
| PC(O-40:6) | 2.37 (1.79,3.17) | 2.00 (1.52,2.51) | 1.95 (1.42,2.53) | 0.309 | 0.995 | 0.221 | 0.29 |
| PC(O-40:7) | 2.25 (1.48,2.92) | 1.92 (1.61,2.37) | 2.04 (1.35,2.57) | 0.592 | 0.971 | 0.758 | 0.552 |
| PC(P-32:0) | 1.76 (1.32,2.10) | 1.42 (1.25,1.74) | 1.53 (1.20,1.90) | 0.091 | 0.766 | 0.554 | 0.213 |
| PC(P-32:1) | 0.256 (0.219,0.335) | 0.223 (0.197,0.271) | 0.231 (0.177,0.277) | **0.038** | 0.991 | 0.221 | 0.128 |
| PC(P-34:1) | 2.65 (2.16,3.34) | 2.13 (1.89,2.64) | 2.35 (1.80,2.75) | **0.017** | 0.99 | 0.113 | 0.07 |
| PC(P-34:2) | 7.89 (5.38,10) | 7.35 (6.29,8.34) | 6.44 (5.17,9.15) | 0.914 | 0.632 | 0.57 | 0.486 |
| PC(P-34:3) | 0.0742 (0.064,0.0951) | 0.0643 (0.0551,0.0819) | 0.0739 (0.0617,0.0919) | 0.149 | 0.328 | 0.905 | 0.217 |
| PC(P-36:2) | 2.45 (2.01,4.26) | 2.63 (2.23,3.27) | 2.29 (1.94,3.44) | 0.999 | 0.771 | 0.863 | 0.701 |
| PC(P-36:4) | 11 (8.43,14) | 11 (8.51,13) | 10 (8.22,12) | 0.992 | 0.933 | 0.953 | 0.856 |
| PC(P-36:5) | 0.706 (0.426,0.991) | 0.487 (0.371,0.842) | 0.495 (0.286,0.757) | 0.483 | 0.607 | 0.107 | 0.201 |
| PC(P-38:4) | 4.46 (2.56,5.65) | 3.86 (3.08,4.93) | 3.57 (2.92,4.85) | 0.971 | 0.971 | 0.968 | 0.877 |
| PC(P-38:5) | 7.35 (5.22,9.51) | 6.61 (5.14,7.51) | 6.20 (4.74,8.69) | 0.388 | 0.999 | 0.453 | 0.403 |
| PC(P-38:6) | 1.36 (0.846,1.96) | 1.23 (0.906,1.69) | 1.11 (0.776,1.87) | 0.8 | 0.979 | 0.651 | 0.601 |
| PC(P-40:6) | 0.997 (0.659,1.31) | 0.912 (0.644,1.13) | 0.795 (0.525,1.10) | 0.728 | 0.799 | 0.393 | 0.416 |
| LPC 14:0 | 2.91 (1.77,3.44) | 2.73 (1.85,3.21) | 1.87 (1.50,2.99) | 0.934 | 0.08 | 0.139 | 0.128 |
| LPC 15:0 | 1.65 (0.991,2.19) | 1.59 (1.13,2.39) | 1.50 (1.10,1.98) | 0.998 | 0.725 | 0.736 | 0.611 |
| LPC 16:0 | 222 (176,283) | 219 (156,273) | 188 (156,232) | 0.926 | 0.55 | 0.211 | 0.322 |
| LPC 16:1 | 5.88 (4.49,6.84) | 5.60 (4.17,7.10) | 4.59 (3.86,6.52) | 0.998 | 0.371 | 0.445 | 0.38 |
| LPC 17:0 | 4.61 (2.70,6.28) | 3.83 (2.51,5.87) | 3.40 (2.26,4.75) | 0.906 | 0.512 | 0.276 | 0.339 |
| LPC 17:1 | 1.05 (0.732,1.30) | 0.993 (0.715,1.37) | 0.867 (0.652,1.20) | 1 | 0.506 | 0.587 | 0.47 |
| LPC 18:0 | 67 (50,86) | 56 (42,81) | 48 (40,61) | 0.455 | 0.185 | **0.004** | **0.035** |
| LPC 18:1 | 40 (33,53) | 39 (30,54) | 34 (30,47) | 0.922 | 0.632 | 0.338 | 0.403 |
| LPC 18:2 | 47 (38,77) | 56 (42,75) | 53 (39,67) | 0.748 | 0.859 | 0.984 | 0.667 |
| LPC 18:3 | 1.18 (0.841,1.78) | 1.19 (0.73,1.66) | 1.00 (0.618,1.34) | 0.998 | 0.475 | 0.554 | 0.447 |
| LPC 20:0 | 0.36 (0.272,0.491) | 0.31 (0.254,0.487) | 0.273 (0.208,0.457) | 0.812 | 0.594 | 0.232 | 0.322 |
| LPC 20:1 | 0.732 (0.537,1.05) | 0.649 (0.434,0.925) | 0.589 (0.418,0.887) | 0.657 | 0.945 | 0.523 | 0.489 |
| LPC 20:2 | 0.712 (0.533,1.04) | 0.647 (0.502,0.956) | 0.599 (0.471,0.919) | 0.947 | 0.743 | 0.476 | 0.49 |
| LPC 20:3 | 7.32 (5.81,10) | 7.00 (5.13,11) | 5.64 (4.09,7.64) | 1 | **0.028** | **0.024** | **0.041** |
| LPC 20:4 | 14 (12,20) | 14 (11,21) | 14 (11,18) | 0.999 | 0.981 | 0.947 | 0.893 |
| LPC 20:5 | 1.57 (0.86,2.13) | 1.00 (0.661,1.65) | 0.832 (0.603,1.27) | 0.314 | 0.626 | **0.039** | 0.12 |
| LPC 22:0 | 0.0674 (0.0527,0.0864) | 0.0638 (0.0518,0.0807) | 0.0568 (0.0429,0.0832) | 0.956 | 0.881 | 0.651 | 0.615 |
| LPC 22:1 | 0.0517 (0.0373,0.0706) | 0.0439 (0.0326,0.0585) | 0.0414 (0.0335,0.063) | 0.401 | 1 | 0.476 | 0.407 |
| LPC 22:5 | 2.06 (1.46,2.50) | 1.60 (1.21,2.37) | 1.57 (1.19,1.93) | 0.281 | 0.962 | **0.046** | 0.154 |
| LPC 22:6 | 3.52 (2.75,6.22) | 3.73 (2.73,5.09) | 3.40 (2.31,4.85) | 0.934 | 0.885 | 0.53 | 0.558 |
| LPC 24:0 | 0.172 (0.132,0.24) | 0.153 (0.121,0.191) | 0.141 (0.116,0.207) | 0.483 | 0.92 | 0.282 | 0.346 |
| LPC 26:0 | 0.0438 (0.0314,0.0568) | 0.0362 (0.0251,0.0492) | 0.0334 (0.0273,0.048) | 0.17 | 0.996 | 0.154 | 0.202 |
| LPC(O-16:0) | 0.986 (0.734,1.45) | 0.884 (0.642,1.16) | 0.912 (0.609,1.10) | 0.526 | 0.967 | 0.288 | 0.376 |
| LPC(O-18:0) | 0.338 (0.249,0.469) | 0.31 (0.22,0.52) | 0.288 (0.225,0.421) | 0.982 | 0.881 | 0.562 | 0.597 |
| LPC(O-18:1) | 0.674 (0.531,0.986) | 0.594 (0.465,0.845) | 0.627 (0.462,0.761) | 0.728 | 1 | 0.713 | 0.615 |
| LPC(O-22:0) | 0.106 (0.0889,0.139) | 0.101 (0.0801,0.132) | 0.0952 (0.0723,0.132) | 0.812 | 0.955 | 0.515 | 0.535 |
| LPC(O-24:0) | 0.191 (0.142,0.229) | 0.16 (0.135,0.206) | 0.139 (0.115,0.192) | 0.428 | 0.298 | **0.029** | 0.087 |
| LPC(O-24:1) | 0.222 (0.194,0.27) | 0.225 (0.181,0.271) | 0.201 (0.166,0.269) | 0.999 | 0.645 | 0.587 | 0.519 |
| LPC(O-24:2) | 0.0167 (0.0138,0.0208) | 0.0139 (0.0103,0.019) | 0.0126 (0.00882,0.018) | 0.221 | 0.942 | 0.175 | 0.221 |
| PE 32:0 | 0.0284 (0.0188,0.0419) | 0.0272 (0.0193,0.0389) | 0.0273 (0.019,0.039) | 0.993 | 1 | 0.995 | 0.971 |
| PE 34:1 | 1.23 (0.882,1.69) | 1.09 (0.795,1.44) | 1.06 (0.786,1.50) | 0.734 | 0.994 | 0.627 | 0.577 |
| PE 34:2 | 1.92 (1.41,3.32) | 2.07 (1.55,3.16) | 1.78 (1.39,2.76) | 0.987 | 0.815 | 0.973 | 0.776 |
| PE 34:3 | 0.114 (0.0667,0.171) | 0.132 (0.0723,0.161) | 0.102 (0.0643,0.131) | 0.953 | 0.494 | 0.919 | 0.537 |
| PE 35:1 | 0.118 (0.0787,0.141) | 0.107 (0.0785,0.171) | 0.0882 (0.0743,0.134) | 0.999 | 0.783 | 0.651 | 0.602 |
| PE 35:2 | 0.119 (0.063,0.172) | 0.111 (0.0691,0.206) | 0.105 (0.0662,0.148) | 0.973 | 0.859 | 0.983 | 0.799 |
| PE 36:0 | 0.0198 (0.0134,0.0245) | 0.017 (0.0137,0.0253) | 0.0191 (0.0131,0.0249) | 0.994 | 1 | 0.998 | 0.979 |
| PE 36:1 | 1.44 (0.975,1.98) | 1.22 (0.964,1.79) | 1.00 (0.741,1.85) | 0.693 | 0.428 | 0.253 | 0.281 |
| PE 36:2 | 6.81 (4.45,10) | 6.64 (5.04,8.94) | 5.49 (3.79,8.48) | 1 | 0.328 | 0.523 | 0.387 |
| PE 36:3 | 2.35 (1.72,3.42) | 2.35 (1.79,3.20) | 2.08 (1.35,2.95) | 0.996 | 0.303 | 0.69 | 0.403 |
| PE 36:4 | 3.60 (2.26,4.66) | 3.09 (2.32,4.46) | 2.91 (2.08,3.91) | 0.941 | 0.676 | 0.499 | 0.479 |
| PE 36:5 | 0.213 (0.126,0.308) | 0.15 (0.124,0.275) | 0.125 (0.0672,0.19) | 0.734 | 0.178 | **0.046** | 0.103 |
| PE 38:3 | 1.02 (0.708,1.36) | 1.03 (0.576,1.41) | 0.601 (0.403,0.943) | 0.975 | **0.013** | **0.005** | **0.022** |
| PE 38:4 | 11 (5.71,14) | 8.28 (6.56,12) | 6.72 (5.45,12) | 0.635 | 0.451 | 0.306 | 0.301 |
| PE 38:5 | 3.49 (1.97,4.35) | 2.76 (1.76,3.88) | 2.38 (1.76,3.28) | 0.768 | 0.55 | 0.175 | 0.281 |
| PE 38:6 | 3.84 (2.05,6.45) | 3.06 (2.12,5.83) | 3.10 (2.15,3.93) | 0.787 | 0.845 | 0.259 | 0.395 |
| PE 40:5 | 1.25 (0.701,1.84) | 0.878 (0.567,1.38) | 0.65 (0.518,1.03) | 0.17 | 0.719 | **0.03** | 0.09 |
| PE 40:6 | 2.36 (1.29,4.90) | 1.91 (1.15,3.24) | 1.88 (1.21,2.46) | 0.65 | 0.859 | 0.242 | 0.356 |
| PE 40:7 | 0.408 (0.264,0.61) | 0.321 (0.225,0.586) | 0.302 (0.197,0.418) | 0.937 | 0.737 | 0.253 | 0.402 |
| PE(O-34:1) | 0.158 (0.121,0.202) | 0.133 (0.0961,0.183) | 0.127 (0.0889,0.155) | 0.139 | 0.998 | 0.06 | 0.133 |
| PE(O-34:2) | 0.15 (0.0966,0.218) | 0.114 (0.08,0.163) | 0.101 (0.0775,0.159) | 0.388 | 0.82 | 0.154 | 0.246 |
| PE(O-36:2) | 0.178 (0.127,0.239) | 0.154 (0.119,0.2) | 0.15 (0.0928,0.205) | 0.414 | 0.933 | 0.242 | 0.318 |
| PE(O-36:3) | 0.221 (0.167,0.298) | 0.177 (0.119,0.236) | 0.175 (0.104,0.221) | 0.203 | 0.976 | 0.104 | 0.181 |
| PE(O-36:4) | 0.894 (0.661,1.15) | 0.774 (0.509,1.06) | 0.677 (0.499,1.05) | 0.519 | 0.955 | 0.379 | 0.403 |
| PE(O-36:5) | 0.0943 (0.0547,0.161) | 0.0551 (0.0334,0.108) | 0.0466 (0.0305,0.0902) | **0.035** | 0.883 | **0.017** | **0.041** |
| PE(O-38:4) | 1.03 (0.767,1.28) | 0.894 (0.64,1.05) | 0.823 (0.506,1.14) | 0.497 | 0.967 | 0.306 | 0.376 |
| PE(O-38:5) | 0.998 (0.77,1.22) | 0.86 (0.598,1.09) | 0.785 (0.567,0.984) | 0.25 | 0.854 | 0.053 | 0.141 |
| PE(O-40:5) | 0.234 (0.143,0.334) | 0.193 (0.143,0.248) | 0.181 (0.127,0.231) | 0.414 | 0.67 | 0.248 | 0.28 |
| PE(O-40:6) | 0.188 (0.138,0.254) | 0.167 (0.103,0.222) | 0.148 (0.11,0.196) | 0.519 | 0.979 | 0.216 | 0.339 |
| Total PE(O) | 4.34 (3.31,4.87) | 3.44 (2.85,4.57) | 3.27 (2.29,4.43) | 0.25 | 0.936 | 0.162 | 0.204 |
| PE(P-34:1) | 0.0929 (0.0566,0.123) | 0.079 (0.0617,0.105) | 0.0759 (0.0601,0.102) | 0.599 | 0.987 | 0.43 | 0.451 |
| PE(P-34:2) | 0.133 (0.0956,0.214) | 0.158 (0.0978,0.204) | 0.131 (0.0832,0.173) | 0.996 | 0.433 | 0.863 | 0.515 |
| PE(P-36:1) | 0.0818 (0.0497,0.11) | 0.0723 (0.046,0.093) | 0.0629 (0.0407,0.0854) | 0.8 | 0.676 | 0.393 | 0.403 |
| PE(P-36:2) | 0.342 (0.225,0.572) | 0.356 (0.274,0.443) | 0.277 (0.199,0.404) | 0.998 | 0.261 | 0.515 | 0.351 |
| PE(P-36:4) | 0.743 (0.407,0.952) | 0.693 (0.494,0.929) | 0.557 (0.426,0.88) | 0.999 | 0.754 | 0.973 | 0.763 |
| PE(P-38:4) | 1.26 (0.584,1.70) | 1.20 (0.875,1.54) | 0.994 (0.759,1.41) | 1 | 0.613 | 0.973 | 0.689 |
| PE(P-38:5) | 1.36 (0.983,1.81) | 1.23 (0.847,1.53) | 1.10 (0.795,1.54) | 0.375 | 0.99 | 0.401 | 0.38 |
| PE(P-38:6) | 0.276 (0.239,0.465) | 0.288 (0.2,0.402) | 0.273 (0.175,0.436) | 0.892 | 1 | 0.869 | 0.788 |
| PE(P-40:5) | 0.502 (0.341,0.604) | 0.427 (0.334,0.526) | 0.426 (0.277,0.528) | 0.533 | 1 | 0.643 | 0.512 |
| PE(P-40:6) | 0.278 (0.207,0.38) | 0.286 (0.201,0.358) | 0.286 (0.142,0.354) | 0.989 | 0.986 | 0.943 | 0.882 |
| LPE 16:0 | 3.03 (2.55,3.93) | 3.20 (2.40,3.95) | 2.89 (2.21,4.42) | 1 | 0.995 | 0.919 | 0.914 |
| LPE 18:0 | 6.33 (4.51,8.83) | 5.56 (4.33,8.18) | 4.50 (3.82,8.38) | 0.857 | 0.845 | 0.468 | 0.491 |
| LPE 18:1 | 4.98 (3.29,6.09) | 5.09 (3.55,6.72) | 4.65 (3.14,6.75) | 0.953 | 0.913 | 0.993 | 0.835 |
| LPE 18:2 | 5.72 (4.44,8.81) | 6.93 (5.41,9.96) | 6.56 (4.98,9.91) | 0.314 | 0.992 | 0.546 | 0.399 |
| LPE 20:4 | 3.75 (3.16,4.61) | 4.29 (3.72,5.48) | 4.29 (3.47,5.23) | 0.23 | 0.962 | 0.445 | 0.32 |
| LPE 22:6 | 2.71 (2.00,4.15) | 2.73 (2.24,4.17) | 2.79 (2.18,4.25) | 0.989 | 0.99 | 0.919 | 0.877 |
| PI 32:0 | 0.668 (0.46,1.26) | 0.683 (0.437,1.04) | 0.525 (0.294,0.803) | 0.926 | 0.284 | 0.126 | 0.204 |
| PI 32:1 | 0.88 (0.453,1.71) | 1.15 (0.559,1.54) | 0.585 (0.422,1.17) | 0.922 | 0.064 | 0.408 | 0.16 |
| PI 34:0 | 0.267 (0.177,0.502) | 0.227 (0.175,0.285) | 0.203 (0.11,0.32) | 0.332 | 0.563 | 0.096 | 0.157 |
| PI 34:1 | 9.63 (6.53,13) | 9.39 (6.18,12) | 6.63 (5.07,8.69) | 0.981 | **0.024** | **0.019** | **0.037** |
| PI 36:1 | 9.08 (6.96,12) | 8.52 (6.36,11) | 6.74 (4.51,8.46) | 0.824 | **0.023** | **0.005** | **0.024** |
| PI 36:2 | 24 (19,38) | 26 (20,32) | 20 (16,30) | 0.973 | 0.371 | 0.338 | 0.335 |
| PI 36:3 | 6.57 (5.56,11) | 7.33 (5.68,10) | 5.42 (4.05,7.59) | 0.991 | **0.023** | 0.086 | 0.063 |
| PI 36:4 | 8.71 (6.09,11) | 8.36 (6.39,9.87) | 6.51 (5.04,8.25) | 0.914 | 0.103 | **0.042** | 0.087 |
| PI 38:2 | 0.699 (0.491,0.969) | 0.59 (0.379,0.693) | 0.42 (0.325,0.646) | 0.099 | 0.371 | **0.006** | **0.034** |
| PI 38:3 | 11 (8.07,15) | 9.65 (7.01,12) | 6.88 (5.27,9.89) | 0.109 | **0.024** | **<0.001** | **0.005** |
| PI 38:4 | 56 (38,74) | 42 (34,59) | 41 (31,55) | 0.132 | 0.965 | 0.119 | 0.157 |
| PI 38:5 | 4.98 (4.06,6.98) | 4.35 (3.35,7.09) | 3.63 (2.55,5.00) | 0.728 | 0.119 | **0.017** | 0.059 |
| PI 38:6 | 1.14 (0.8,2.07) | 1.24 (0.929,1.68) | 0.907 (0.639,1.24) | 1 | **0.015** | **0.04** | **0.04** |
| PI 40:4 | 0.822 (0.526,1.17) | 0.611 (0.484,0.901) | 0.557 (0.436,0.867) | 0.135 | 0.897 | 0.086 | 0.137 |
| PI 40:5 | 2.49 (1.57,3.23) | 2.02 (1.33,2.42) | 1.56 (1.27,1.96) | 0.132 | 0.275 | **0.008** | **0.035** |
| PI 40:6 | 1.86 (1.01,3.34) | 1.65 (1.32,2.55) | 1.59 (1.08,2.09) | 0.995 | 0.563 | 0.635 | 0.503 |
| LPI 18:0 | 1.77 (1.40,2.62) | 1.70 (1.18,2.08) | 1.81 (1.20,2.11) | 0.592 | 0.998 | 0.793 | 0.585 |
| LPI 18:1 | 1.23 (0.904,1.56) | 1.41 (1.05,1.79) | 1.02 (0.733,1.33) | 0.533 | **0.011** | 0.276 | 0.052 |
| LPI 18:2 | 1.49 (0.959,1.83) | 1.81 (1.33,2.59) | 1.26 (0.988,1.87) | 0.101 | 0.098 | 0.999 | 0.123 |
| LPI 20:4 | 1.59 (1.12,1.96) | 1.82 (1.31,2.19) | 1.40 (1.10,1.86) | 0.592 | 0.223 | 0.959 | 0.322 |
| PS 36:1 | 2.44 (1.02,7.71) | 1.57 (0.884,6.77) | 1.82 (0.623,6.83) | 0.592 | 0.991 | 0.779 | 0.574 |
| PS 38:4 | 2.54 (1.13,8.67) | 1.51 (1.03,7.38) | 2.00 (0.663,6.88) | 0.504 | 0.999 | 0.611 | 0.489 |
| PS 40:6 | 0.267 (0.114,0.598) | 0.158 (0.0826,0.316) | 0.142 (0.0841,0.518) | 0.35 | 0.994 | 0.69 | 0.445 |
| COH | 1352 (1073,1783) | 1133 (911,1353) | 1028 (835,1318) | 0.065 | 0.794 | **0.008** | **0.039** |
| CE 14:0 | 24 (14,35) | 21 (15,28) | 15 (12,23) | 0.714 | 0.219 | **0.049** | 0.115 |
| CE 15:0 | 22 (18,29) | 20 (14,30) | 18 (15,27) | 0.714 | 0.707 | 0.143 | 0.284 |
| CE 16:0 | 508 (433,582) | 420 (357,524) | 423 (376,487) | **0.031** | 0.999 | **0.008** | **0.037** |
| CE 16:1 | 107 (76,183) | 100 (65,149) | 92 (69,134) | 0.835 | 0.93 | 0.43 | 0.498 |
| CE 16:2 | 3.60 (2.75,4.77) | 2.95 (2.05,4.89) | 2.39 (1.84,3.97) | 0.714 | 0.323 | **0.044** | 0.128 |
| CE 17:0 | 11 (8.71,15) | 9.13 (6.47,15) | 9.17 (6.74,11) | 0.614 | 1 | 0.423 | 0.466 |
| CE 17:1 | 44 (39,52) | 37 (30,48) | 35 (31,43) | 0.129 | 0.962 | **0.015** | 0.073 |
| CE 18:0 | 32 (28,39) | 28 (22,34) | 26 (24,34) | 0.069 | 0.984 | **0.04** | 0.085 |
| CE 18:1 | 269 (234,333) | 231 (181,265) | 231 (190,284) | **0.013** | 1 | **0.017** | **0.035** |
| CE 18:2 | 282 (241,346) | 238 (203,312) | 232 (215,290) | 0.074 | 0.994 | **0.016** | 0.063 |
| CE 18:3 | 43 (31,50) | 34 (23,50) | 27 (21,39) | 0.584 | 0.144 | **0.005** | **0.037** |
| CE 20:1 | 0.336 (0.291,0.448) | 0.277 (0.225,0.348) | 0.292 (0.225,0.375) | **0.023** | 0.939 | 0.088 | 0.07 |
| CE 20:2 | 1.78 (1.30,2.16) | 1.35 (0.962,1.86) | 1.05 (0.853,1.33) | 0.159 | **0.038** | **<0.001** | **0.005** |
| CE 20:3 | 26 (20,31) | 21 (17,27) | 21 (16,25) | 0.132 | 0.936 | **0.035** | 0.102 |
| CE 20:4 | 146 (92,167) | 100 (85,146) | 102 (81,120) | 0.123 | 0.985 | 0.057 | 0.123 |
| CE 20:5 | 31 (15,55) | 17 (11,33) | 15 (9.36,22) | 0.139 | 0.457 | **0.009** | **0.041** |
| CE 22:0 | 0.213 (0.131,0.313) | 0.171 (0.139,0.251) | 0.15 (0.115,0.259) | 0.781 | 0.575 | 0.253 | 0.322 |
| CE 22:1 | 0.11 (0.0984,0.215) | 0.0992 (0.0777,0.131) | 0.101 (0.0695,0.13) | 0.074 | 0.897 | **0.047** | 0.087 |
| CE 22:4 | 0.097 (0.0711,0.126) | 0.0771 (0.0612,0.106) | 0.08 (0.0665,0.0984) | 0.174 | 0.997 | 0.119 | 0.184 |
| CE 22:5 | 1.50 (1.11,2.21) | 1.07 (0.746,1.53) | 0.909 (0.76,1.24) | **0.012** | 0.754 | **<0.001** | **0.006** |
| CE 22:6 | 12 (8.88,31) | 11 (8.46,19) | 11 (6.95,14) | 0.49 | 0.575 | 0.122 | 0.207 |
| CE 24:1 | 0.157 (0.117,0.211) | 0.124 (0.0951,0.151) | 0.11 (0.076,0.146) | **0.018** | 0.825 | **0.007** | **0.027** |
| CE 24:5 | 0.00762 (0.00519,0.0107) | 0.00672 (0.00521,0.00895) | 0.00612 (0.00446,0.00848) | 0.941 | 0.463 | 0.345 | 0.362 |
| DG 14:0/18:1 | 1.57 (0.946,2.22) | 1.21 (0.875,2.25) | 0.75 (0.475,1.20) | 0.962 | **0.003** | **0.005** | **0.014** |
| DG 14:0/18:2 | 0.58 (0.452,1.17) | 0.612 (0.313,1.06) | 0.354 (0.263,0.599) | 1 | **0.013** | **0.021** | **0.034** |
| DG 16:0/18:1 | 16 (11,21) | 13 (8.30,22) | 9.76 (7.88,16) | 0.707 | 0.298 | **0.033** | 0.113 |
| DG 16:0/20:3 | 0.422 (0.243,0.568) | 0.357 (0.197,0.542) | 0.232 (0.173,0.33) | 0.918 | **0.018** | **0.008** | **0.025** |
| DG 16:0/20:4 | 0.792 (0.46,1.40) | 0.76 (0.502,1.18) | 0.546 (0.36,0.844) | 0.993 | 0.109 | 0.158 | 0.156 |
| DG 16:0/22:5 | 0.328 (0.216,0.571) | 0.303 (0.175,0.499) | 0.233 (0.176,0.345) | 0.734 | 0.289 | 0.056 | 0.135 |
| DG 16:0/22:6 | 0.311 (0.123,0.695) | 0.288 (0.164,0.536) | 0.198 (0.097,0.46) | 1 | 0.382 | 0.523 | 0.403 |
| DG 18:0/18:2 | 1.51 (0.908,2.36) | 1.20 (0.764,2.21) | 1.08 (0.609,1.66) | 0.824 | 0.376 | 0.086 | 0.184 |
| DG 18:0/20:4 | 0.467 (0.255,0.631) | 0.363 (0.253,0.516) | 0.319 (0.207,0.639) | 0.555 | 0.916 | 0.46 | 0.429 |
| DG 18:1/18:1 | 19 (13,27) | 16 (11,25) | 12 (9.57,17) | 0.606 | 0.239 | **0.014** | 0.067 |
| DG 18:1/18:2 | 17 (8.02,23) | 14 (8.80,20) | 11 (7.63,16) | 0.873 | 0.284 | 0.062 | 0.154 |
| DG 18:1/18:3 | 2.58 (1.83,3.69) | 1.83 (1.39,3.51) | 1.51 (1.10,1.96) | 0.768 | **0.044** | **0.002** | **0.022** |
| DG 18:1/20:3 | 1.19 (0.812,1.70) | 1.01 (0.594,1.35) | 0.739 (0.516,0.938) | 0.761 | **0.027** | **0.002** | **0.019** |
| DG 18:1/20:4 | 2.66 (1.73,4.45) | 2.14 (1.60,4.24) | 1.86 (1.48,2.68) | 0.852 | 0.117 | **0.024** | 0.07 |
| DG 18:2/18:2 | 3.27 (1.74,4.45) | 2.91 (1.40,5.40) | 2.54 (1.33,4.20) | 1 | 0.664 | 0.603 | 0.535 |
| TG 14:0/16:0/18:1 | 16 (9.20,22) | 13 (8.71,24) | 8.43 (5.03,16) | 0.967 | **0.044** | **0.039** | 0.062 |
| TG 14:0/16:0/18:2 | 6.54 (3.99,10) | 5.70 (3.60,9.50) | 3.95 (2.30,7.87) | 0.953 | 0.135 | 0.154 | 0.159 |
| TG 14:0/16:1/18:1 | 13 (8.34,20) | 9.56 (6.41,17) | 5.81 (3.10,12) | 0.664 | **0.015** | **0.007** | **0.022** |
| TG 14:0/16:1/18:2 | 3.28 (2.08,5.27) | 3.08 (1.56,4.96) | 1.66 (1.06,2.99) | 0.93 | **0.007** | **0.005** | **0.018** |
| TG 14:0/17:0/18:1 | 3.02 (1.96,4.04) | 2.85 (2.15,4.62) | 2.26 (1.40,3.43) | 1 | 0.235 | 0.237 | 0.258 |
| TG 14:0/18:0/18:1 | 0.594 (0.453,0.888) | 0.533 (0.392,0.697) | 0.376 (0.249,0.528) | 0.847 | **0.008** | **0.011** | **0.022** |
| TG 14:0/18:2/18:2 | 1.74 (1.08,2.49) | 1.68 (0.936,3.26) | 1.18 (0.668,1.62) | 1 | **0.048** | **0.029** | 0.06 |
| TG 14:1/16:0/18:1 | 2.08 (1.31,3.24) | 1.61 (1.08,2.97) | 1.05 (0.639,2.43) | 0.714 | 0.172 | 0.113 | 0.141 |
| TG 14:1/18:0/18:2 | 0.436 (0.314,0.548) | 0.354 (0.271,0.523) | 0.253 (0.18,0.381) | 0.533 | **0.023** | **0.003** | **0.018** |
| TG 14:1/18:1/18:1 | 6.51 (4.41,8.83) | 5.87 (3.92,9.23) | 4.16 (2.98,5.66) | 0.981 | **0.03** | **0.011** | **0.035** |
| TG 15:0/16:0/18:1 | 2.82 (1.86,3.56) | 2.28 (1.72,3.74) | 1.62 (1.03,3.16) | 0.988 | 0.122 | 0.136 | 0.154 |
| TG 15:0/18:1/18:1 | 0.992 (0.838,1.24) | 1.06 (0.744,1.34) | 0.813 (0.526,1.20) | 1 | 0.289 | 0.188 | 0.258 |
| TG 16:0/16:0/18:0 | 7.08 (4.79,9.43) | 5.99 (4.64,11) | 5.44 (4.11,9.41) | 0.944 | 0.62 | 0.445 | 0.441 |
| TG 16:0/16:0/18:1 | 59 (39,80) | 47 (37,90) | 41 (31,78) | 0.902 | 0.626 | 0.401 | 0.413 |
| TG 16:0/16:0/18:2 | 14 (9.30,20) | 13 (8.29,20) | 13 (7.74,17) | 0.947 | 0.885 | 0.578 | 0.585 |
| TG 16:0/16:1/17:0 | 3.27 (2.17,4.37) | 2.88 (2.07,5.11) | 2.33 (1.47,4.30) | 1 | 0.531 | 0.437 | 0.431 |
| TG 16:0/16:1/18:1 | 65 (51,96) | 52 (39,82) | 45 (29,65) | 0.533 | 0.235 | **0.046** | 0.102 |
| TG 16:0/17:0/18:0 | 0.219 (0.144,0.265) | 0.169 (0.101,0.261) | 0.15 (0.0903,0.265) | 0.755 | 0.825 | 0.365 | 0.417 |
| TG 16:0/17:0/18:1 | 2.30 (1.66,3.03) | 2.24 (1.40,3.33) | 1.69 (1.16,2.85) | 0.993 | 0.457 | 0.175 | 0.304 |
| TG 16:0/17:0/18:2 | 3.94 (3.10,4.90) | 3.48 (2.53,5.46) | 2.90 (2.11,4.78) | 0.93 | 0.575 | 0.162 | 0.31 |
| TG 16:0/18:0/18:1 | 19 (12,21) | 14 (11,20) | 14 (11,19) | 0.441 | 0.994 | 0.188 | 0.317 |
| TG 16:0/18:1/18:1 | 135 (92,162) | 103 (77,149) | 101 (80,144) | 0.388 | 0.989 | 0.179 | 0.292 |
| TG 16:0/18:1/18:2 | 82 (46,106) | 65 (45,96) | 67 (50,89) | 0.748 | 0.969 | 0.507 | 0.516 |
| TG 16:0/18:2/18:2 | 17 (11,23) | 15 (8.36,22) | 13 (9.53,19) | 0.873 | 0.982 | 0.658 | 0.64 |
| TG 16:1/16:1/16:1 | 1.18 (0.571,1.71) | 0.888 (0.537,1.66) | 0.608 (0.395,1.13) | 0.975 | **0.04** | 0.053 | 0.067 |
| TG 16:1/16:1/18:0 | 0.833 (0.546,1.07) | 0.75 (0.493,1.11) | 0.541 (0.356,0.706) | 0.982 | **0.016** | **0.009** | **0.027** |
| TG 16:1/16:1/18:1 | 7.05 (4.21,12) | 5.46 (3.80,9.14) | 5.19 (3.63,7.90) | 0.897 | 0.731 | 0.3 | 0.403 |
| TG 16:1/17:0/18:1 | 6.59 (5.58,7.97) | 6.44 (4.33,8.18) | 5.26 (3.45,7.07) | 0.977 | 0.371 | 0.154 | 0.258 |
| TG 16:1/18:1/18:1 | 8.34 (5.70,12) | 7.24 (4.76,10) | 6.63 (4.90,7.79) | 0.8 | 0.607 | 0.143 | 0.281 |
| TG 16:1/18:1/18:2 | 18 (11,24) | 16 (9.87,23) | 14 (9.56,17) | 0.847 | 0.531 | 0.083 | 0.221 |
| TG 17:0/18:1/18:1 | 4.88 (3.64,6.04) | 4.15 (2.98,5.97) | 3.65 (2.95,4.90) | 0.526 | 0.777 | 0.083 | 0.213 |
| TG 18:0/18:0/18:1 | 1.63 (0.924,2.60) | 1.23 (0.808,2.98) | 0.958 (0.543,1.72) | 0.868 | 0.219 | 0.074 | 0.144 |
| TG 18:0/18:1/18:1 | 14 (8.90,20) | 12 (8.65,16) | 9.31 (6.99,14) | 0.657 | 0.215 | **0.049** | 0.11 |
| TG 18:0/18:2/18:2 | 9.40 (6.21,14) | 8.49 (5.72,15) | 7.98 (4.30,12) | 0.988 | 0.737 | 0.476 | 0.505 |
| TG 18:1/18:1/18:1 | 23 (16,32) | 19 (15,27) | 18 (13,27) | 0.812 | 0.766 | 0.3 | 0.4 |
| TG 18:1/18:1/18:2 | 18 (9.50,22) | 16 (11,23) | 15 (8.49,20) | 1 | 0.676 | 0.674 | 0.57 |
| TG 18:1/18:1/20:4 | 1.57 (1.09,2.11) | 1.34 (0.922,2.14) | 1.27 (1.04,1.72) | 0.818 | 0.771 | 0.253 | 0.38 |
| TG 18:1/18:1/22:6 | 1.71 (1.30,2.78) | 1.74 (1.22,2.33) | 1.66 (1.16,2.10) | 0.969 | 0.804 | 0.423 | 0.498 |
| TG 18:1/18:2/18:2 | 9.42 (5.82,14) | 7.74 (5.34,15) | 7.25 (3.90,11) | 0.988 | 0.626 | 0.453 | 0.461 |
| TG 18:2/18:2/18:2 | 1.04 (0.542,1.56) | 0.943 (0.542,1.86) | 0.853 (0.418,1.72) | 1 | 0.82 | 0.923 | 0.769 |
| TG 18:2/18:2/20:4 | 0.418 (0.3,0.644) | 0.366 (0.251,0.768) | 0.382 (0.206,0.497) | 0.999 | 0.575 | 0.562 | 0.489 |

**Supplementary Material Table 1 Footnote:**

Medians and interquartile ranges (IQR)

**^a^** Computed using Mann-Whitney U test, and corrected by Dunn-Sidak method

^b^ Computed using Kruskal-Wallis test and corrected using Benjamini Hochberg method

**Abbreviations:** Ceramide (**Cer**), Monohexosylceramide (**MHC**), Dihexosylceramide (**DHC**), Trihexosylceramide (**THC**), GM3 ganglioside (**GM3**), Sphingomyelin (**SM**), Phosphatidylcholine (**PC**), Alkylphosphatidylcholine (**PC-O**), Phosphatidylcholine plasmalogen (**PC-P**), Lysophosphatidylcholine (**LPC**), Lysoalkylphosphatidylcholine (**LPC(O)**), Phosphatidylethanolamine (**PE**), Akylphosphatidylethanolamine (**PE-O**), Phosphatidylethanolamine plasmalogen (**PE-P**), Lysophosphatidylethanolamine (**LPE**), Phosphatidylinositol (**PI**), Lysophosphatidylinositol (**LPI**), Phosphatidylserine (**PS**), Free Cholesterol (**COH**), Cholesterol Ester (**Ce**), Diacylglycerol (**DG**), Triacylglycerol (**TG**).
